# Investigation of the Flexibility of Protein Kinases Implicated in the Pathology of Alzheimer’s Disease

**DOI:** 10.3390/molecules19079134

**Published:** 2014-06-30

**Authors:** Michael P. Mazanetz, Charles A. Laughton, Peter M. Fischer

**Affiliations:** Centre for Biomolecular Sciences and School of Pharmacy, University of Nottingham, University Park, Nottingham NG7 2RD, UK; E-Mails: charles.laughton@nottingham.ac.uk (C.A.L.); peter.fischer@nottingham.ac.uk (P.M.F.)

**Keywords:** active site pressurisation, kinase, structure-based drug design, protein flexibility, molecular dynamics

## Abstract

The pathological characteristics of Alzheimer’s Disease (AD) have been linked to the activity of three particular kinases—Glycogen Synthase Kinase 3β (GSK3β), Cyclin-Dependent Kinase 5 (CDK5) and Extracellular-signal Regulated Kinase 2 (ERK2). As a consequence, the design of selective, potent and drug-like inhibitors of these kinases is of particular interest. Structure-based design methods are well-established in the development of kinase inhibitors. However, progress in this field is limited by the difficulty in obtaining X-ray crystal structures suitable for drug design and by the inability of this method to resolve highly flexible regions of the protein that are crucial for ligand binding. To address this issue, we have undertaken a study of human protein kinases CDK5/p25, CDK5, ERK2 and GSK3β using both conventional molecular dynamics (MD) and the new Active Site Pressurisation (ASP) methodology, to look for kinase-specific patterns of flexibility that could be leveraged for the design of selective inhibitors. ASP was used to examine the intrinsic flexibility of the ATP-binding pocket for CDK5/p25, CDK5 and GSK3β where it is shown to be capable of inducing significant conformational changes when compared with X-ray crystal structures. The results from these experiments were used to quantify the dynamics of each protein, which supported the observations made from the conventional MD simulations. Additional information was also derived from the ASP simulations, including the shape of the ATP-binding site and the rigidity of the ATP-binding pocket. These observations may be exploited in the design of selective inhibitors of GSK3β, CDK5 and ERK2.

## 1. Introduction

AD is the most common cause of dementia in older people and affects more than 30 million people worldwide [1,2]. It is a degenerative disease of the brain resulting in cognitive and behavioural impairment. Two of the neuropathological hallmarks for AD, in addition to severe brain atrophy and neuronal loss, are the formation of dense extra-cellular deposits and intracellular aggregates within neurons, which have been identified as amyloid (neuritic) plaques and neurofibrillary tangles, respectively [3,4,5,6,7,8,9]. The tangles are the result of the misfolding of hyperphosphorylated tau [7] the major microtubule-associated protein in the axons of neurons in vertebrate brains.

Although these pathological hallmarks of plaques, tangles and neuronal death all contribute to the aetiology of AD, the mechanistic relationships between these defining lesions remain to be determined. However, growing evidence indicates that inhibition of tau hyperphosphorylation is a viable therapeutic strategy for AD. There are a number of protein kinases which phosphorylate tau *in vitro*. However, only a small number of these have been implicated in tau phosphorylation *in vivo*, and few have been linked to abnormal tau hyperphosphorylation [10]. The three that have received the most attention are GSK3β, CDK5 and ERK2 [11]. Evidence in the literature has implicated GSK3β and CDK5 in the *in vivo* and *in vitro* regulation of tau phosphorylation and association with microtubules [12,13]. ERK2 has been found to be deregulated in post mortem AD brains, and there is evidence that ERK2 may be a requirement in neurofibrillary degeneration from the observation that ERK is dysregulated as a likely result of oxidative stress [14].

**Figure 1:**
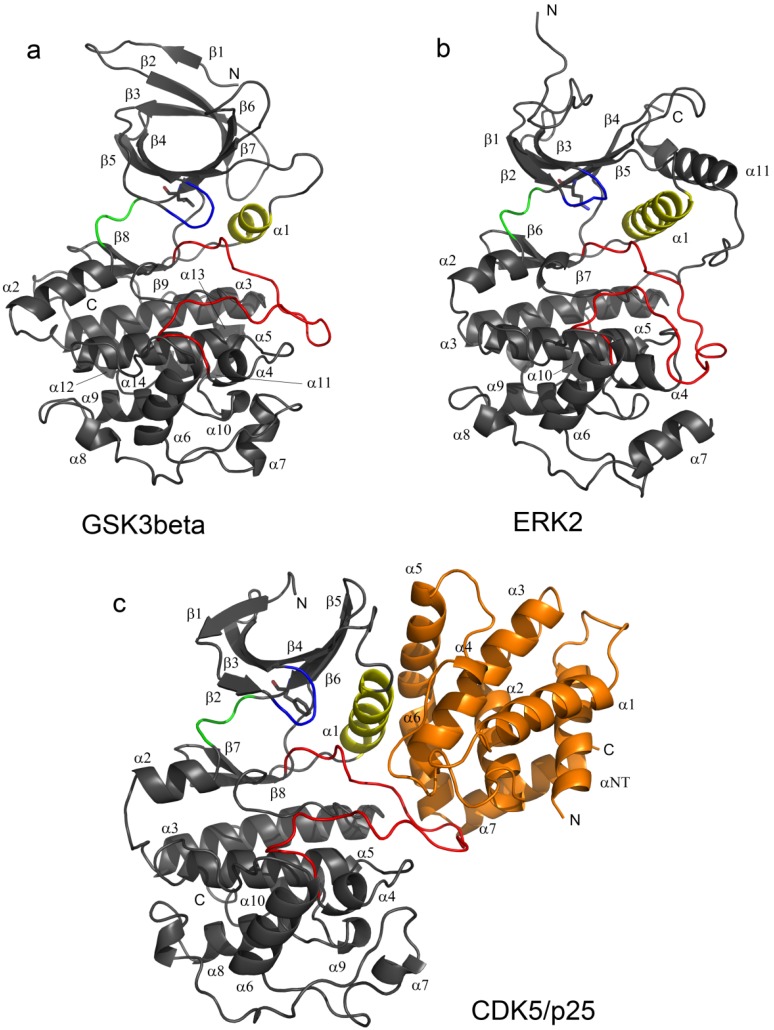
Kinases are shown as secondary structure cartoons representations (grey). Common structural features are colour coded as follows: PSTAIRE/helix αC, yellow; activation loop (T-loop), red; hinge region, green; Gly-rich loop (P-loop), blue. The gate-keeper residue in represented in CPK (Corey-Pauling-Koltun) sticks. The three modelled structures are (**a**) GSK3β (**b**) ERK2 and (**c**) CDK5/p25, p25 depicted in orange.

CDK5, GSK3β, and ERK2 are phylogenetically and structurally closely related kinases of the CMGC family [11]. They share high sequence homology and display similar structural elements that are involved in the regulation of their activity [11]. These three kinases show the typical two-lobed globular kinase fold with a β-strand domain at the N-terminus and an α-helical domain at the C-terminus [15] (see Figure 1).

MD simulations have been used previously to examine ligand-protein interactions for CDK5, GSK3β and ERK2 [16,17,18,19,20,21]. Much of this work has been focused on specific ligand-protein interactions in an effort to understand biological activity in the absence of X-ray crystal structures. Results have highlighted the need to consider solvation during the simulations [17,20,21]. Molecular modelling of CDK5 and CDK5/p25 bound with small molecule inhibitors have helped define the molecular interactions required for ligand binding, and how dynamics could explain the involvement of the regulatory subunit p25 in CDK5 activity [19,22]. Results from MD simulations on two GSK3β-ligand complexes showed that P-loop flexibility is important in forming H-bonding interactions with the ligands bound in the ATP-binding sites [17].

**Figure 2:**
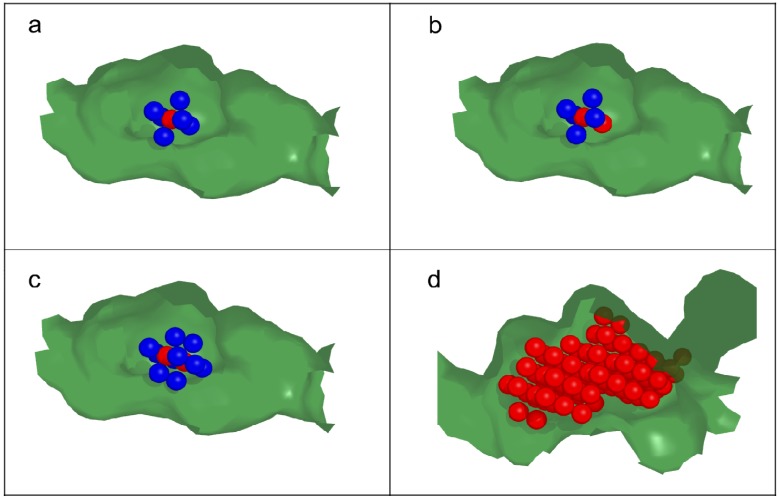
Active Site Pressurisation methodology. (**a**) The first particle in the cast is centred in a binding site and activated (red). The surrounding latent particles (blue) are arranged on a cubic grid and are held in fixed space; (**b**) A simulation of normal protein movement is performed over a short time period. The forces acting on latent particles adjacent to the activated central grid particle are accumulated. The latent particle experiencing the lowest force is activated and becomes part of the cast; (**c**) The simulation is restarted and the process is repeated; (**d**) Over a number of iterations the grid particles in the catalytic site expand in a direction that exploits the greatest flexibility in the protein structure and creates an optimal cast.

These results from the literature exemplify the general observation that a knowledge of protein dynamics and flexibility is a major advantage, and sometimes a requirement, for the understanding of molecular recognition events. A variety of computational and theoretical methods have been developed to help with this, including statistical thermodynamics, [23] graph theory, [24] constraint theory, [25] geometric constraints, [26] elastic networks, [27] graph theory combined with elastic networks, [28] and knowledge-based energy functions [29]. The most convenient and extensively studied method to characterise protein flexibility is MD simulation. We have developed a novel MD-based method for examining protein flexibility [30] and have used it to rationalise protein-ligand binding [31,32]. This method, called Active Site Pressurisation (ASP), predicts energetically favourable pathways through which binding sites in proteins may distend to accommodate bound ligands. ASP was originally implemented using a modified version of AMBER [7,33] and a new version has since been implemented in MOE [34]. In ASP, uncharged Lennard-Jones particles (termed ASP particles) in the form of a *resin* are injected into a binding site to produce a *cast* of the shape of the active site. Then, more ASP particles can be forced into the active site under a controllable pressure, determined by the force acting upon the *resin*, causing the active site to distend and the entire protein to deform in an energetically favorable manner. A conceptual representation of the steps performed during the *grid* version of the ASP method are detailed in Figure 2.

**Figure 3:**
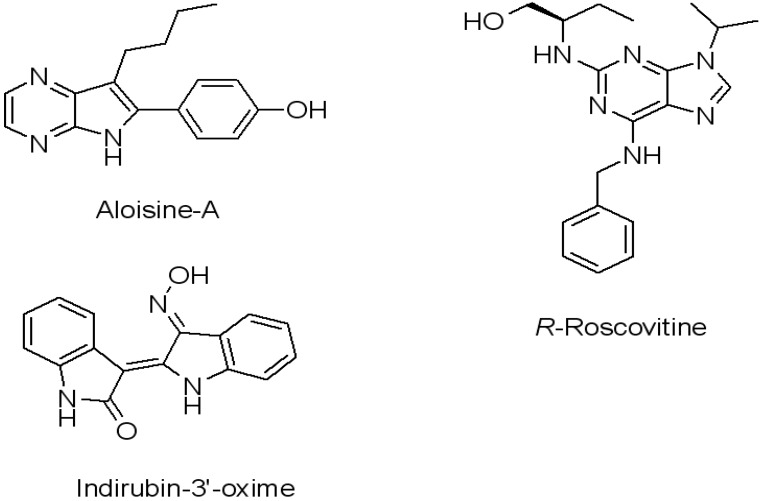
Structures of aloisine-A, indirubin-3'-oxime, *R*-roscovitine used in the study.

Herein we describe a study of the human protein kinases using conventional MD simulations on four CDK5^D144N^/p25 complexes with the ligands *R*-roscovitine, aloisine-A, indirubin-3'-oxime and ATP, see Figure 3. The ligands were selected as they have been well characterised in the literature both in terms of their activity against CDK5, GSK3 and ERK2 and also the availability of X-ray structural data, *vide infra* [11]. These results were compared to simulations of the apo CDK5/p25 structure. In turn, the results from these simulations were compared to those of GSK3β and ERK2. Finally, the intrinsic flexibility of the ATP-binding sites of CDK5, CDK5/p25 and GSK3β, were examined using the ASP method. The results from these experiments have been processed to yield a quantitative and comparative analysis of the dynamics of each protein. Such information could potentially be used to guide the structure-based design of potent and selective kinase inhibitors.

## 2. Results and Discussion

### 2.1. Structural and Dynamics Analysis

**Figure 4:**
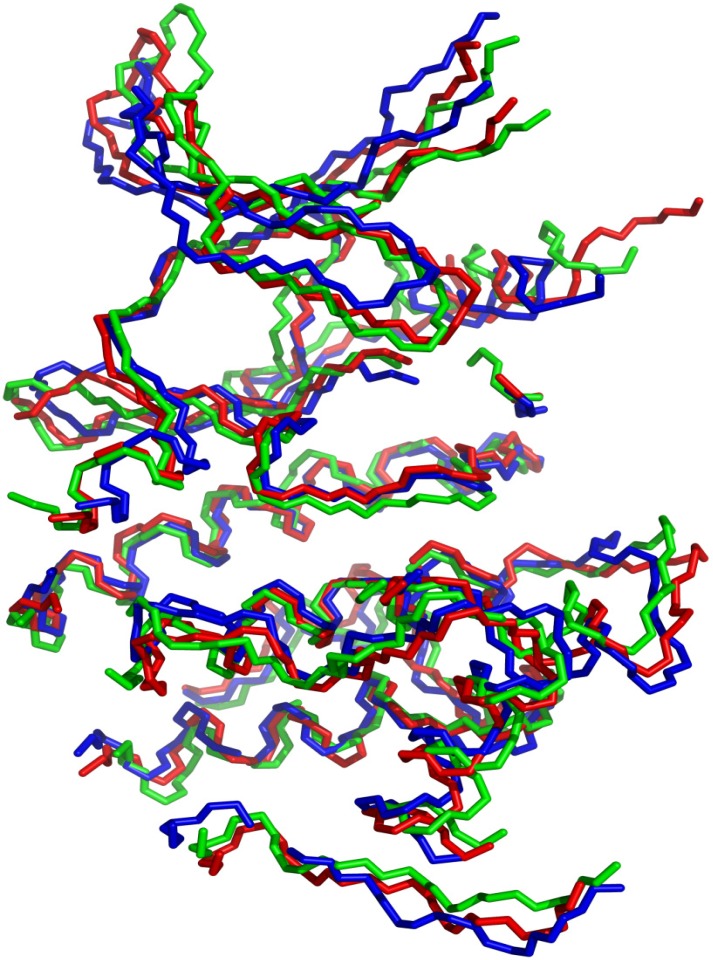
Backbone (C_α_, C, N) of ERK2 (blue), GSKβ (red) and CDK5 (green) included in the trajectory analysis.

MD simulations were completed on protein structures of mutant CDK5^D144N^/p25 in multiple complexes (including the apo structure and bound with ATP/Mg^2+^, indirubin-3'-oxime, *R*-roscovitine and aloisine-A). Additional simulations were performed on CDK5/p25, GSK3β and ERK2. In an effort to discriminate between the proteins of interest on the basis of the movement of the backbone atoms, atomic fluctuations were determined from the final 3.5 ns of each production run. The protein models were aligned using PyMOL (see Figure 4), [35] and the highly conserved structural regions dictated by overlap of the backbone atoms (C_α_, C, and N), referred to as the *core*, were used to compare the proteins, see Table 1 and Table 2. This was necessary as the systems need to contain the same atoms in order to be comparable for principal component analysis (PCA) and structural analysis of the MD trajectories (see the PCA section of Protein Dynamics).

**Figure 5:**
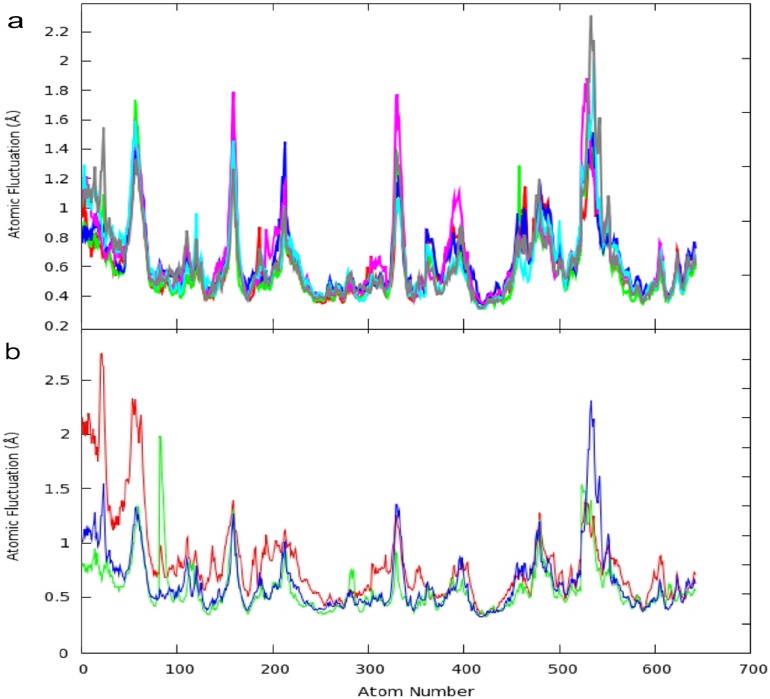
(**a**) Atomic fluctuations (Å) in the *core* of 642 atoms during the final 3.5 ns of the 6 CDK5 MD simulations. Graph shows the atomic fluctuations (Å) of the C, C_α_ and N atoms in the backbone during the last 3.5 ns of the MD simulations for: CDK5^D144N^/p25 bound with aloisine-A (magenta), indirubin-3'-oxime (red), *R*-roscovitine (green) and ATP (blue); apo CDK5^D144N^/p25 (grey) and apo CDK5/p25 (cyan); (**b**) Atomic fluctuations (Å) in the *mask* of 642 atoms during the final 3.5 ns of the GSK3β (green), ERK2 (red) and CDK5/p25 (blue) MD simulations.

Analysis of the MD simulations for the six CDK5 models shows that the systems share very similar atomic fluctuations (see Figure 5a). The average standard deviation between the atoms within the *core* for these structures is 0.072 Å. Observations were made on the core atoms, see Figure 5a. The majority of the atomic fluctuations of the residues which constitute the ATP binding pocket does not vary greatly between the apo and the ligand bound CDK5^D144N^/p25 structures. 

**Table 1:** Details of the 214 residues which made up the *core* as used for CDK5.

Residue Numbers	Number of Amino Acids	*Core* Atom Numbering
7L-33K	27	1–81
47S-72S	26	82–159
75K-92D	18	160–213
98L-144D	47	214–354
147L-148A	2	355–360
166W-197N	32	361–456
199G-220G	22	457–522
241M-250N	10	523–552
255L-284P	30	553–642

**Table 2:** RMSD (Å) between the CDK5^D144N^/p25 model and the other kinase models studied for the residues within the *core*.

Protein	RMSD (Å)	Residues in *core*
CDK5^D144N^/p25	0.000	7–33, 47–72, 75–92, 98–144, 147–148, 166–197, 199–220, 241–250, 255–284
GSK3β	1.164	35–61, 69–94, 103–120, 129–167, 169–176, 179–180, 197–250, 270–279, 285–314
ERK2	1.184	21–47, 59–64, 66–85, 93–110, 114–160, 163–164, 185–238, 260–266, 268–270, 275–304

**Figure 6:**
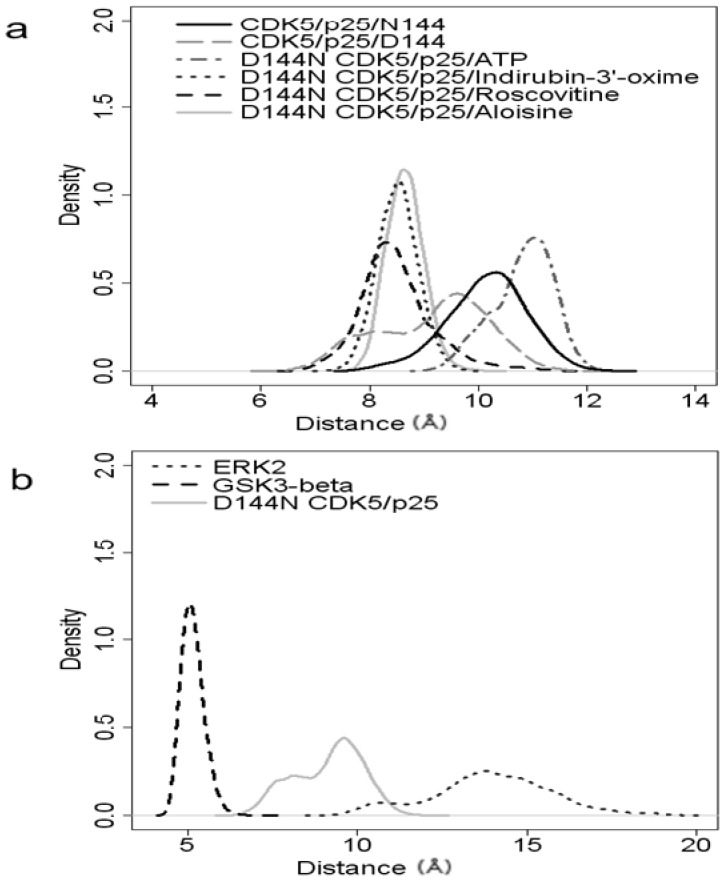
Probability distributions for the distances: (**a**) between the center of mass of Thr14 and Gly146 in CDK5 structures; and (**b**) between the center of mass of Ala28 and Gly162 in ERK2, Ser42 and Gly178 of GSK3β and between the center of mass of Thr14 and Gly146 in CDK5.

The α-helical motifs, α1 (atoms 82–114), α3 (atoms 221–282) and α5 (atoms 407–453) are all relatively stable with RMSDs of 0.55 Å, 0.463 Å and 0.42 Å, respectively. The hinge region (atoms 175–195) has little mobility, RMSD of 0.522 Å, and the DFG motif (atoms 352–354) does not fluctuate significantly (RMSD of 0.441 Å). The most flexible binding pocket feature is the P-loop (RMSD of 0.898 Å). This degree of flexibility is conserved amongst the CDK5/p25 structures examined. The flexibility of the P-loop has also been studied by Zhang *et al*., for the complex of *R*-roscovitine and CDK5^D144N^ and the apo structure with and without p25 [19]. In this account the authors measured the distance between the C_α_ of Thr14 in the P-loop to the C_α_ of Gly146 in the DFG motif. The average distance between these atoms for the ligand bound structure was 7.3 Å, with an approximate range of ±1 Å. Our own analysis from the center of mass of these two residues gave a comparable average distance of 8.49 Å with an approximate range of ±2 Å. These results are comparable for the other ligands examined, see Figure 6a. The ligands induce some stability into the P-loop compared to the two apo structures of CDK5/p25 and CDK5^D144N^/p25 which show a greater degree of motion within the P-loop, with standard devations of 1.02 and 0.74 respectively. It is interesting to note that the ATP-binding site is distended to the greatest degree by the binding of ATP/Mg^2+^ out of the four ligands examined. The average distance was 10.81 Å. This is markedly different to the average distance of 5.2 Å observed by Zhang *et al*., for the apo CDK5^D144N^ structure. Thus there is an observable opening of the P-loop following activation of CDK5.

The distances between the center of mass of Thr14 and the center of mass of Gly146 for the other ligand complexes *R*-roscovitine, indirubin and aloisine-A were 8.49 Å, 8.51 Å and 8.65 Å, and with standard deviations of 0.68, 0.58 and 0.34, respectively. The range of motion observed in the *R*-roscovitine-bound complex was the largest compared to the other liganded complexes as shown by the probability distributions in Figure 6a.

Other more flexible protein loops include the loop between β3 and β4 (atoms 49–60) (RMSD 1.247 Å), the loop joining β5 and β6 (atoms 115–141) (RMSD 1.30 Å), and the loop between α6 to α8 (atoms 520–549) (RMSD 1.108 Å) in the C-terminal alpha helical domain are all very mobile, see Figure 5a.

The RMSDs between the initial starting structures and the time averaged structures for aloisine-A, indirubin-3'-oxime, *R*-roscovitine and the ATP/Mg^2+^ system were 1.168 Å, 1.672 Å, 1.035 Å and 1.398 Å, respectively. An examination of the RMSD of the CDK5^D144N^/p25 model bound with the ligands at the start of the MD simulation and the time averaged structure, together with the atomic fluctuations data, shows that the effect of a ligand bound within the ATP-binding site on the flexibility of the protein is very subtle. These observations justify the use of the CDK5^D144N^ mutant in the protein modelling using molecular mechanics.

The atomic fluctuations for the aligned backbone atoms of GSK3β, ERK2 and CDK5/p25 were compared, see Figure 5b. These results show that conserved regions between GSK3β and CDK5/p25 are very similar in both the degree and range of motion, the average standard deviation between the atoms within the *core* is 0.08. However, ERK2 is slightly less similar, the average standard deviation between the atoms within the *core* for these structures is 0.163.

In all of the MD simulations, the largest atomic fluctuations (between 0.8 and 2.8 Å) in the conserved regions in the *core* were observed between the Gly-rich P-loop (β1, β2 and β3, atoms 1–66), the residues which form a loop between β4 and β5 (atoms 155–164), the residues which form a loop between β7 and β8 (atoms 326–338) and α6 to α8 (atoms 450–580) (as per the CDK5 secondary structural numbering convention), see Figure 1

These results were not unexpected as protein function and specificity is associated with the variable loop regions such as the P-loop, *vide infra*. Of particular note were the differences observed with ERK2, where the atomic fluctuations are greater than 1 Å above those observed for the other kinase systems studied, see Figure 5b. For example the ERK2 Gly-27 backbone contained within the P-loop has an atom fluctation of approximately 1.5 Å greater than that of either CDK5 or GSK3β. The greater flexibility in the Gly-rich P-loop of ERK2 compared with CDK5 and GSK3β could explain why ligands which have a twisted, non-planar conformation are more specific for ERK2 [11]. The reduced flexibility in the P-loop in CDK5 and GSK3β could account for smaller more planar molecule inhibitors showing selectivity for these kinases over ERK2. This can be observed in the average distance the P-loop moves relative to the DFG mofit during the MD similations. This distance was 5.16 Å for GSK3β, 9.15 Å for CDK5/p25 and 13.93 Å for ERK2, see Figure 6b.

The MD simulations performed shed some light on the flexibility of the protein kinases, and the extent to which ligands can affect protein conformation. The atomic fluctuations observed indicate that ERK2 has a significantly different degree of protein flexibility than CDK5 or GSK3β. Therefore, further studies on ERK2 were not undertaken as distinguishable dynamics for ERK2 were already identified. The question remains whether the latter two kinases can be distinguished based on their inherent flexiblity.

### 2.2. Analysis of Active Site Pressurisation Dynamics

To examine inherent protein flexibility further, the ASP methodology was applied to CDK5, CDK5/p25 and GSK3β to address two questions. Firstly, starting from apo-type conformations, could ASP generate biologically relevant ligand-binding conformations and demonstrate the particular flexibility of the ATP-binding site as seen in X-ray crystal structures? Secondly, could ASP predict novel accessible receptor conformations that might be useful in drug design? The protocol for the *grid* version of ASP was followed. ASP particles were inserted (on a 1.1 Å grid) into the binding site of GSK3β, CDK5 and CDK5/p25 over a 400 ps time scale.

**Figure 7:**
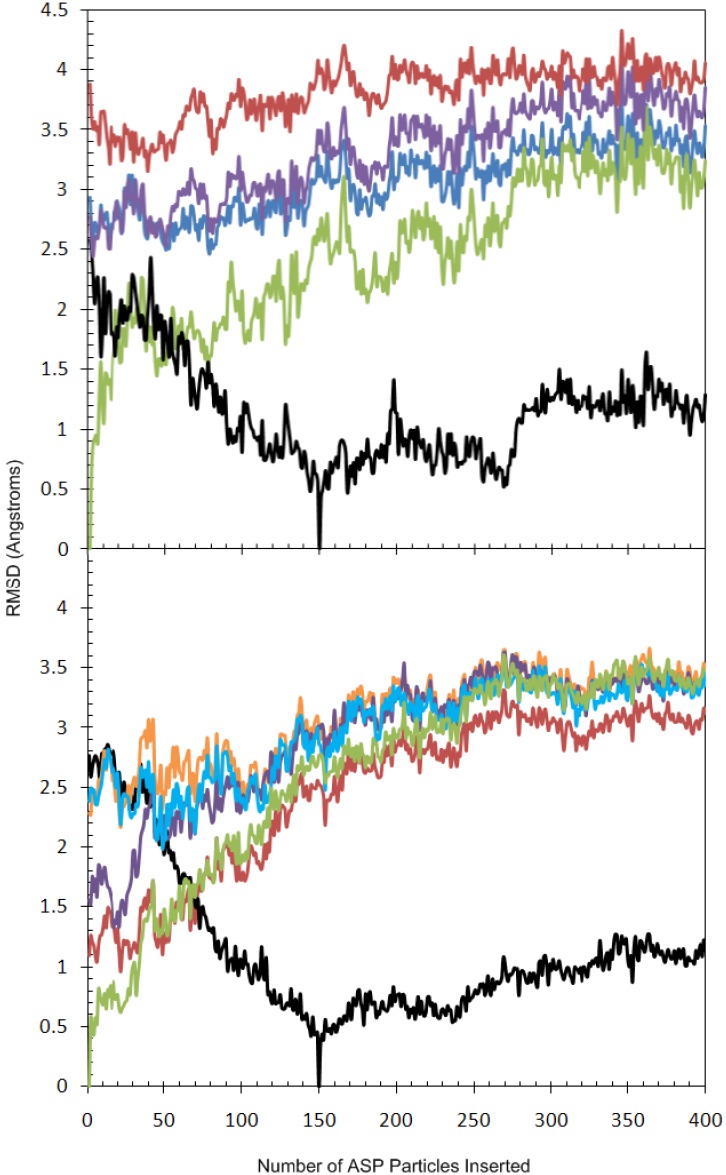
(**a**) Evolution of the RMSD of the backbone atoms of the Gly-rich P-loop (residues 37–47) of GSK3β during the ASP simulation for 1.1 Å-spaced ASP particles, with respect to reference structures. Reference structures included the protein conformations containing 1 ASP particle (green), those containing 150 ASP particles (black), and three X-ray crystal structures for GSK3β: 1Q3W (blue), 1Q4L (purple), and 1R0E (red); (**b**) Evolution of the RMSD of the backbone atoms of key residues defining the CDK5 binding pocket including the residues 11–17 (the Gly-rich P-loop), 31, 33, 80–85 (the Hinge region), 130, 133 and 144–146 (the DFG motif) of CDK5 during the ASP simulation for 1.1 Å-spaced ASP particles with respect to reference structures. Reference structures included the protein conformations containing 1 ASP particle (green), those containing 150 ASP particles (black), and four ligand complexes taken from the MD simulations: *R*-roscovitine (red), aloisine-A (orange), indirubin-3'-oxime (purple) and ATP/Mg^2+^ (cyan).

Previously, we reported how conformational changes observed in the Gly-rich P-loop (residues 37–47) of GSK3β across the ASP MD trajectory samples conformations that are significantly close to those observed in the crystal structures of a variety of ligand complexes [30]. Herein we report a detailed comparison of the motions of the Gly-rich P-loop during the ASP simulation to the initial protein conformation at the start of the ASP simulation (when 1 particle is activated), after 150 ASP particles were activated, and to three X-ray crystal structures taken from the PDB (1Q3W, 1Q4L and 1R0E), [36] see Figure 7a.

The changes can be described as taking place in four stages. Stage 1; the activation of the first 40 ASP particles fills the space in the binding cavity, as noted by the rapid increase in RMSD. The protein conformation changes from the apo form to a conformation that is similar to that observed in 1Q4L. Thus, the initial Gly-rich P-loop conformation has an RMSD of 3.88 Å from that observed in the crystal structure of the protein complexed with an indolylarylmaleimide inhibitor (PDB code 1R0E), but after 39 ASP particles were added, this is reduced to 3.15 Å. Stage 2; further addition of ASP particles induces conformational changes in the flexible residue side chains containing freely rotatable bonds. A gradual increase in RMSD of 2.4 Å during the insertion of 40–150 ASP particles is observed. By the time 53 ASP particles has been added, the Gly-rich P-loop conformation had adjusted further to more closely resemble that observed in the structures of GSK3β as the phosphorylated complex and the inhibitor bound form (PDB codes 1I09 and 1H8F [37] respectively, RMSD reductions of 0.61 and 0.68 Å). Finally, when 79 ASP particles has been added, the Gly-rich P-loop reached a conformation considerably closer to that observed in the complex with staursporine (PDB code 1Q3D) than is the case in the original model (RMSD reduced from 2.79 Å to 2.21 Å). Stage 3; after about 150 ASP particles are activated, a point is reached where the ASP particles no longer have an effect on the protein conformation and instead expand in the direction of the solvent. This is shown by the average change in RMSD of 0.8 Å for the protein conformation during the insertion of 150–250 ASP particles. Stage 4; once the ASP particles have filled the solvent volume defined by the ASP box settings, the particles begin to expand the protein further and this is translated into an increase in the RMSD of 0.4 Å for the two ASP-related protein conformations during the activation of 270–310 ASP particles. The RMSD remains relatively constant after this point as the ASP particles fill a volume that does not affect protein conformation within the Gly-rich P-loop.

The movement in the P-loop is in agreement with the proposed mechanism of kinase function [38]. The P-loop conformation is known to shift from a closed state in the apoenzyme to a more open state upon ATP binding, this is also confirmed by the MD simulations for CDK5, see Figure 7b. However, conformational changes different to those typically observed upon ATP binding were also evident. Distinct pockets in and around the ATP-binding site opened up in response to the growing mass of ASP particles, and as a consequence residues distal to the ATP-binding site also underwent conformational change by as much as 7.5 Å from their original positions as a result of ASP. For example, in the process of inserting 150 ASP particles a cleft opened close to the opening of the pocket near residue Arg117, and a second pocket at the rear of the pocket close to residues Ala59, Ile60 and Lys61.

We were interested in comparing the movements of the P-loop to that of known X-ray structures for CDK5. However, there are only 3 X-ray crystal structures containing ligands within the public domain, of which none has complete residue chains within 8 Å of the bound ligand. Therefore the comparison of RMSDs between the ASP results for CDK5/p25 and the CDK5 X-ray crystal structures was not investigated. Instead the RMSDs of backbone (N, C_α_, and C atoms) of key residues within the ATP-binding site during the course of the ASP simulation were compared to the average conformations generated from the MD simulations for the CDK5/p25 ligand complexes, see Figure 7b. The following residues were included in this examination; 11–17 of the P-loop, 31, 33, 80–85 of the Hinge region, 130, 133 and 144–146 of the DFG mofit. During the ASP simulation for CDK5/p25 the ATP-binding site moves progressively further away from the initial insertion of 1 ASP particle to a maximum of 3.61 Å. This expansion is in response to the increasing number of ASP particles filling the ATP-binding site. The process can be examined in stages similar to the GSK3β example. Stage 1; the activation of the first 43 ASP particles fills the space in the binding cavity, as noted by the rapid increase in RMSD to 1.71 Å. At this point there is an increase in the size of the pocket by 0.82 Å compared to the pocket conformation after 43 particles were inserted. This is the largest conformation change upon the addition of one ASP particle during the 400 particle insertion (data not shown). During this stage the ASP distended pocket more closely resembles the inhibitor bound conformations. After 17 ASP particles are inserted the volume is similar to the indirubin-3'-oxime MD simulation averaged structure with and RMSD of 1.32 Å. After 22 ASP particles are inserted the pocket is most similar to the *R*-roscovitine MD simulation averaged structure with an RMSD of 0.96 Å. The aloisine-A MD simulation averaged structure is less similar with an RMSD of 2.16 Å. Stage 2; further addition of ASP particles induces conformational changes in the flexible residue side chains in the P-loop. A gradual increase in RMSD to 2.77 Å during the insertion of 44–147 ASP particles is observed. At the time 49 ASP particles were added, the Gly-rich P-loop conformation has adjusted further to more closely resemble that observed in the MD averaged structure of ATP bound CDK5^D144N^/p25, with a reduction in RMSD from 2.39 to 1.99 Å at the start of the simulation. Stage 3; after about 150 ASP particles are activated, the pocket slowly expands to accommodate the increasing number of particles, and the average change between additions of particles is ±0.45 Å. This is shown by the constant increase in RMSD during the insertion of about 150–240 ASP particles. In the process of inserting 150 ASP particles a well defined pocket opened near the gate-keeper residue Phe80 and the surrounding residues Glu51, Leu55, Ala143 and Phe145. Stage 4; There is a further subtle increase in the size of the pocket once around 250 ASP particles have been inserted. The ATP-binding site continues to expand uniformly, and unlike in GSK3β there is no expansion into the solvent and therefore a gradual increase in pocket volume is observed during this period.

**Figure 8:**
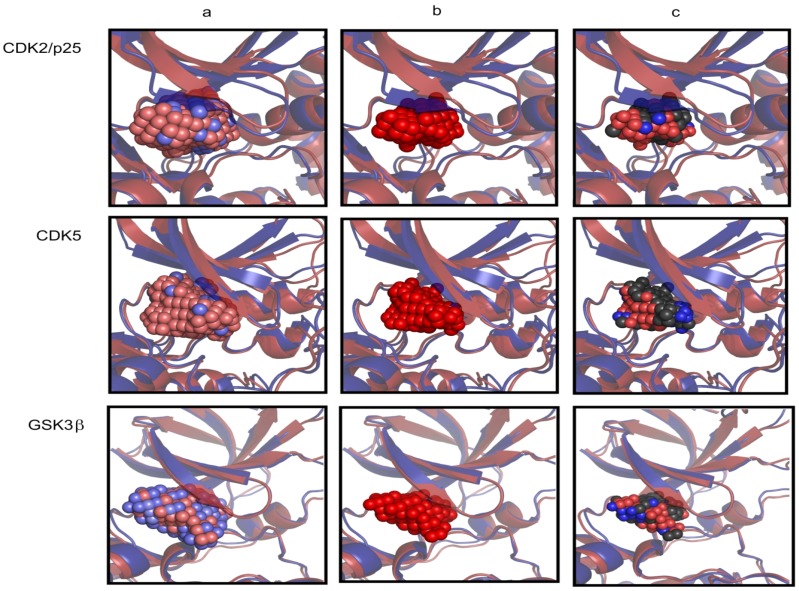
The results of the ASP MD simulation illustrated for CDK5/p25, CDK5 and GSK3β. Graphic showing the starting protein conformation prior to ASP (blue cartoon model) and the conformation after the insertion of 150 ASP particles (red cartoon model). (**a**) The latent ASP particles coloured from high force (blue spheres) to low force (red spheres); (**b**) The kinase protein conformations after insertion of 150 ASP particles are depicted similarly in red including a depiction of the 150 activated ASP particles (red spheres); (**c**) A depiction of the 150 activated ASP particles coloured according to the nearest heavy atom neighbour of the protein (CPK coloured spheres).

An examination of the protein conformations at different stages of the ASP simulation further supports the RMSD results, see Figure 8. The Gly-rich P-loop of CDK5/p25 expands upwards to a greater degree than for the uncomplexed CDK5 structure. This can be understood in terms of the activation loop of CDK5, which is in the unactivated conformation in the uncomplexed form leading to a closed binding pocket, and in the p25 complexed form, the loop is more than 25 Å away and this complex is in the ATP-binding conformation. To accommodate the expanding ASP particle mass in the CDK5 system, the protein distends the β2 and β3 strands, and expands upwards in the direction of the hinge region.

Detailed information about the plasticity of the ATP-binding sites can be extracted from the ASP simulations. The direction in which the ASP particle ensembles expand during the ASP MD simulation to create the binding site cast is a direct result of the plasticity of the protein. The ASP particles are only added to the growing mass in the direction of minimal force acting on the particles (as measured on the latent particles). A pictorial representation of the cast of 150 ASP particles for CDK5/p25, CDK5 and GSK3β is shown in Figure 8.

The *latent* ASP particles are illustrated as a sphere representation, coloured according to the magnitude of the force imposed on each of the latent ASP particles by the surrounding protein. This illustration helps to explain the observed direction of growth of the ASP particle mass during the simulation. This mass grows where there are low force constraints, thus reducing steric clashes with neighbouring protein atoms. High force constraints correlate with the degree of overlap of van der Waals radii with neighboring protein atoms and the ASP particles.

The ASP simulation for GSK3β displays a large number of high force spheres compared to either the CDK5 or CDK5/p25 ASP results, particularly towards the surface specific residues at the opening of the binding site. The casts created by the ASP particles result in geometrically distinct moulds of the ATP-binding sites. The CDK5 casts are more globular in shape than the GSK3β cast, which is longer and flatter, as supported by the measurements between the DFG motif and the P-loop in the MD simulations.

### 2.3. Principal Component Analysis of Protein Dynamics

It is important in analysing an ASP simulation to discriminate between conformational changes that are driven by the process of pressurisation, and those that are due to normal thermal fluctuations. PCA should help in distinguishing between these two types of motion. PCA provides a useful means of analysing molecular dynamics simulations of biomolecules, by reducing the dimensionality of the system [39,40,41]. The largest eigenvectors from PCA describe the most significant large scale concerted atomic motions in the protein. It is possible to compare the vectors generated from one simulation with another, allowing quantitative assessment of the similarity between the dynamics in two different systems [42,43,44]. Providing the two systems have the same number of atoms, and have been fitted to the same reference structure, a measure of the similarity of two individual vectors is supplied by the inner product.

**Figure 9:**
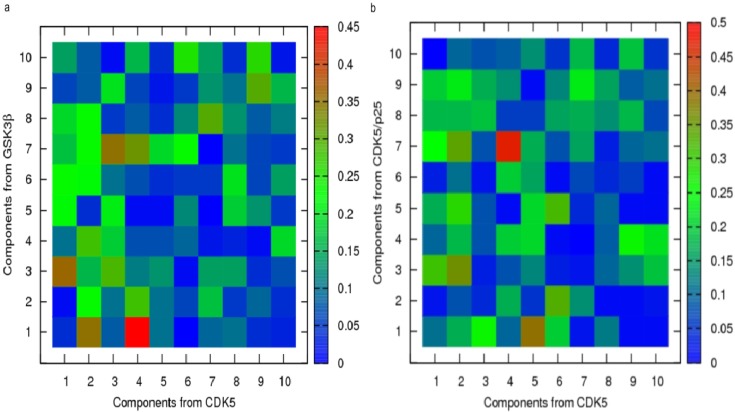
Inner product comparisons for the top ten ranking eigenvectors from simulations of (**a**) CDK5 and GSK3β; (**b**) CDK5 and CDK5/p25. Red squares signify a high level of similarity between two vectors, green for an intermediate level and blue for poor or no similarity.

**Figure 10:**
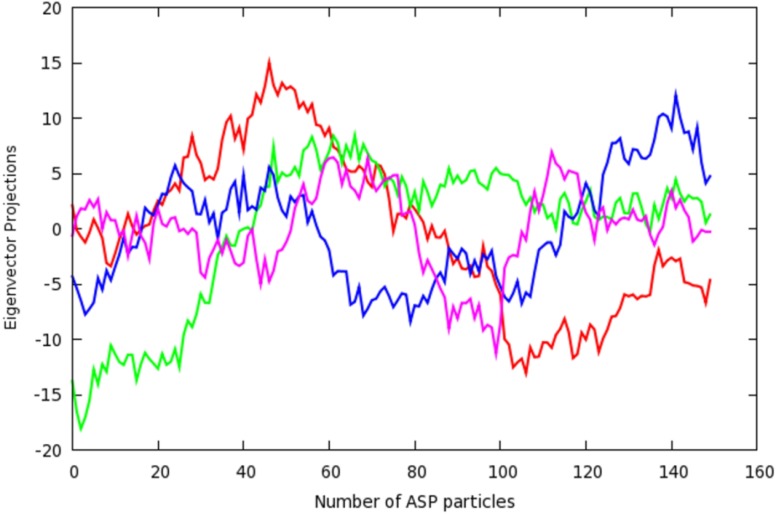
Projections of the first 4 eigenvectors for the PCA analysis of CDK5 for the insertion of 150 ASP particles. The graphs are coloured: eigenvector 1 (red), eigenvector 2 (green), eigenvector 3 (blue), eigenvector 4 (magenta).

To probe the origins of this similarity between the ASP simulations, and to identify the contributing individual dynamic modes, it was necessary to calculate the inner product of each individual pair of vectors. The result of this analysis (a dot product matrix) is displayed in Figure 9 in a graphical format. The most significant similarity observed between the CDK5/p25 dynamics and those of CDK5 is found when comparing mode 4 from the CDK5 simulation with mode 7 from that of CDK5/p25. These two modes show a high degree of similarity, with the red square, and an inner product of 0.47. An examination of the projections of the first four eigenvectors for the PCA analysis of the ASP simulation of CDK5 show that the second component gives rise to a non-oscillating feature that is likely to be a result of the effects from the ASP method, see Figure 10. For CDK5/p25, a similar non-harmonic curve was observed for component 1 (results not shown). The inner product of the two modes associated with ASP induced dynamics was 0.17. This result demonstrates that ASP is affecting these proteins in markedly different ways, and that similar motion is largely accounted for by normal dynamics.

Examination of Figure 9a, shows that for GSK3β and CDK5 the ASP induced modes have a degree of dissimilarity, with an inner product of 0.35. Interestingly, the component 4 of CDK5 which reflects normal dynamics, is similar to the dynamics observed in GSK3β as a result of ASP.

**Figure 11:**
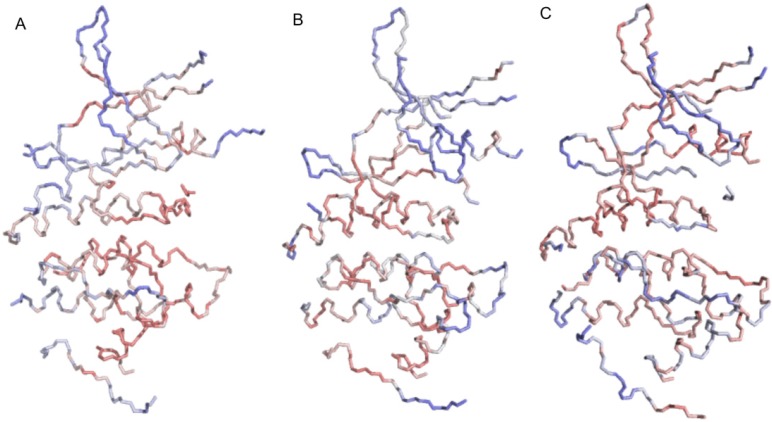
Atomic fluctuations associated with the eigenvector likely to result from the ASP method mapped onto the *mask* atoms (as detailed in Table 1 and Table 2) for (**a**) GSK3β; (**b**) CDK5; and (**c**) CDK5/p25. Colour gradient ramps from red (low atomic fluctuation) through white (medium atomic fluctuation) to blue (high atomic fluctuation).

To understand which motions are a result of the effects of ASP during the CDK5/p25, CDK5 and GSK3β simulations, the atomic fluctuations for the associated components were examined, see Figure 11. The largest atomic fluctuations in CDK5/p25 (0.080–0.174 Å) are within the loop between β7 and β8, in β4, α7 and the Gly-rich P-loop. The largest atomic fluctuations observed for CDK5 (0.078–0.091 Å) were also in the loop between β7 and β8, in the α7, in the Gly-rich P-loop, and in the loop between α2 and α3. The largest atomic fluctuations for GSK3β (0.090–0.154 Å) was within the backbone atoms of the Gly-rich P-loop and the PSTAIRE α-helix.

## 3. Experimental

### 3.1. Ligand Parameterisation

Optimised structures and electrostatic potentials for the ligands were calculated using quantum mechanics at the HF/6-31G* level using GAUSSIAN 98 [45]. Non-standard amino acid residues and ligands not described in the parameterisation libraries in LEaP [46] were parameterised using the AMBER utility *antechamber* [47] and general AMBER force field (GAFF), [48] see the supporting information. ATP parameters were taken from the literature [49]. Ligand parameterisations were checked against the quantum mechanics calculations by *in vacuo* MD simulations with *sander* [50,51].

Following molecular mechanics parameterisation and successful validation, the ligands were manually docked back into the crystal structures using the molecule superimposition tools in CHIMERA [52]. Docking poses were based on the original binding geometry or from similarity to known poses from homologous protein sequences.

### 3.2. Molecular Dynamics Simulations

Proteins were parameterised in LEaP using the AMBER 2003 (Duan ff03) force field [53]. The complexes were neutralised by addition of an appropriate number of counter ions, either sodium or chloride, and immersed in a truncated octahedral box of around 14,000 pre-equilibrated TIP3P water molecules [54,55]. Each water box extended 8 Å away from any solute atom. At each stage, parameter, topology and coordinate files were saved. The cut-off distance for non-bonded interactions was 10 Å. Periodic boundary conditions were applied and electrostatic interactions were represented using the smooth PME method, [56] with constant volume conditions applied. Minimisations were performed using the *sander* module of AMBER 8.0 package [57]. The simulation protocol involved initial solvent and ion density equilibration and minimisation. A total of 1000 minimisation steps were performed; initially 500 steps of steepest descent, followed by 500 steps of conjugate gradient minimisation. The cut-off distance for non-bonded interactions was 10 Å and a force constant of 500 kcal/mol/Å^2^ was used to restrain the proteins. The entire system was then subjected to 2500 steps of minimisation without restraints, initially 1,000 steps of steepest descent, followed by 1500 steps of conjugate gradient minimisation.

This was followed by a 20 ps heating phase, before running a 700 ps equilibration and 3.5 ns of production runs. This 3.5 ns unrestrained trajectory was simulated at 300 K temperature and 1 atm pressure. SHAKE [58] was applied to all bonds involving hydrogen atoms, allowing an integration step of 2 fs. Analysis of trajectories was done with the *ptraj* module of AMBER. After re-centering the coordinates to origin and re-imaging the trajectories to the primary box and after stripping off all the water molecules and counter ions, the last 3.5 ns of trajectory (7000 frames in total) was used to calculate the average coordinates and for further analysis.

PCA of the production trajectories were examined using *ptraj* module of AMBER. The time course of the protein backbone (N, C_α_, C, and O atoms) trajectory projections along the first ten eigenvectors of its covariance matrix across the simulation showed that the systems had approached convergence (results not shown). The contributions of the first 10 modes to the overall motion of the backbone atoms accounted for more than 90% of the motion of the protein systems in all cases and demonstrated that the production MD runs sampled the systems well.

Protein models were built from X-ray structures deposited in the PDB [59]. The protein FASTA [60] sequences were obtained from the PDB for the protein of interest. Then, using the BLAST [61,62] program from the NCBI-BLAST webpage, [63] pairwise sequence-sequence comparisons between the target sequence and available template structure sequences from a variety of databases were performed with the BLOSUM-62 substitution matrix [64]. The crystal structures chosen were then subject to multiple sequence alignment using either the MatchMaker [65] tool in CHIMERA using both the Needleman-Wunch algorithm [66] and the BLOSUM-62 matrix, or by using CLUSTALW [67] through the Biology Workbench web pages [63,68]. The 3D coordinates for template structures were obtained using the alignment tools in PyMOL [35]. The alignments and the associated 3D coordinates in PDB format were used to obtain 3D models using the MODELLER 8v2 program [69].

### 3.3. Protein Models

The PDB contains X-ray crystal structures of CDK5/p25 bound to aloisine-A (PDB code 1UNG), *R*-roscovitine (PDB code 1UNL) and indirubin-3'-oxime (PDB code 1UNH), see Table 3. These ligands are complexed to mutant CDK5^D144N^. The structure of the wild-type protein is available in the apo form (PDB code 1H4L), Table 3, yet the enzymatically inactive mutant structure has formed the basis for previous SAR studies in the literature [70]. The apo form also contains a second mutation, A199G, located on the loop between α5 and α6 which is distal (>15 Å) from the ATP-binding site. The target of the structure-based drug design strategy against CDK5 is the wild-type protein, therefore MD simulations of proteins containing both Asp144 and Asn144 were performed and the results compared to determine whether these structures are significantly different.

**Table 3:** Crystal structures used in homology model building detailing the indicators of refinement associated with each structure. ^a^ NA indicates that the data was not available.

PDB Code	Kinase	R Factor	R*^free^* Factor ^a^	Resolution (Å)	Ramachandran Analysis		Reference
					Favoured Regions (98%)	Allowed Regions (>99.8%)	
1UNH	CDK5	0.229	0.230	2.35	93.9	98.9	[70]
1UNL	CDK5	0.216	0.219	2.20	95.0	98.9	[70]
1UNG	CDK5	0.216	0.225	2.30	91.7	98.1	[70]
1H4L	CDK5	0.236	0.287	2.65	89.0	97.2	[71]
1JST	CDK2	0.200	NA	2.60	92.7	98.4	[72]
1PW2	CDK2	0.213	0.249	1.95	97.9	99.3	[73]
1Q4L	GSK3β	0.212	0.251	2.77	94.3	98.2	[36]
1I09	GSK3β	0.242	0.274	2.70	91.9	98.8	[74]
1Q5K	GSK3β	0.222	0.242	1.94	94.2	98.4	[75]
1TVO	ERK2	0.263	0.272	2.50	90.5	97.1	[76]

**Figure 12:**
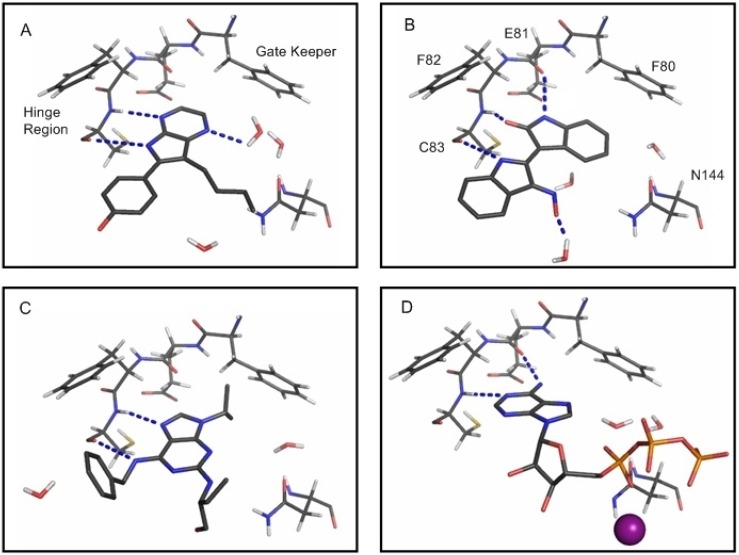
Depiction of the main interactions formed between the ligand (sticks), waters (sticks) and residues (lines) in the ATP-binding site of the CDK5^D144N^/p25 mutant. (**a**) Aloisine-A; (**b**) indirubin-3'-oxime; (**c**) *R*-roscovitine; (**d**) ATP and Mg^2+^. The dashed lines (blue) indicate H-bonding between the ligand and the surrounding protein and water molecules. The gate-keeper residue, F80, is shown in (**a**) and the residues which make up the hinge region in CDK5.

The mutant CDK5^D144N^/p25 model was constructued from the X-ray structures 1UNL and 1H4L, Table 3. The CDK5/p25 model was built using the mutant CDK5^D144N^/p25 model and Asp144 residue coordinates were taken from CDK2 (PDB code 1JST), Table 3. The coordinates for docking ATP and Mg^2+^ were determined by aligning the known chain A from the CDK2/cyclin A/ATP/Mg^2+^ structure (PDB code 1JST) with chain A from the modelled structure of CDK5/p25. Two water molecules H-bonded to ATP in the 1JST structure were also included in the CDK5/p25 model. The final model of CDK5^D144N^/p25 together with bound ATP and Mg^2+^ and the two water molecules is shown in Figure 12d.

The QM optimised geometry of aloisine-A was realigned to the ligand in the PDB file 1UNG. Also included from this PDB file were 3 water molecules bound within 6 Å of the ligand. The final model of CDK5^D144N^/p25 together with bound aloisine-A and the three water molecules is shown in Figure 12a. Similarly, the QM-optimised geometry of indirubin-3'-oxime was realigned to the ligand in the PDB file 1UNH. Also included from this PDB file were four water molecules bound within 6 Å of the ligand. The final model of CDK5^D144N^/p25 together with bound indirubin-3'-oxime and the three water molecules is shown in Figure 12b. The optimised geometry of *R*-roscovitine was realigned to the ligand in the PDB file 1UNL. Also included from this PDB file were two water molecules bound within 6 Å of the ligand. The final model of CDK5^D144N^/p25 together with bound *R*-roscovitine and the two water molecules is shown in Figure 12c.

CDK5 was modelled on the X-ray crystal structure of the human CDK2 apoenzyme (PDB code 1PW2), see Table 3. The GSK3β model was built from three structures (1I09, 1Q4L and 1Q5K) and the ERK2 model was built from 1TVO [76].

### 3.4. Active Site Pressurisation

A conceptual representation of the steps performed in an ASP MD simulation is shown in Figure 2. The protocol for setting up and performing the ASP MD simulations followed the methodology detailed previously [30].

### 3.5. Grid Orientation and Dimensions

The center of the ATP binding site was determined using MOLCAD [77] and a cubic grid box was then constructed around this point which was composed of ASP particles which had the default equilibrium distance of 1.4 Å and with a box volume between 3375 Å^3^ and 8000 Å^3^. This volume was sufficiently large to envelope the area of interest, such that ASP particles would not reach the box boundary before the simulation was deemed to be complete. The orientation of the grid box relative to the protein of interest was adjusted using functions in PyMOL. The program LEaP was employed to generate the parameter, topology, coordinate, structure, and library files for these ASP particle grids.

### 3.6. ASP MD Method

Energy minimisation and MD simulations were performed using the *sander* module of the AMBER 7 package modified to incorporate the *grid* version of ASP. ASP particles (assigned a mass of 10 Da) were restrained to their positions on a 1.1 Å spaced grid using a force constant of 0.1 kcal/mol. The simulation protocol involved initial solvent and ion optimisation and density equilibration. A total of 1000 minimisation steps were performed; initially 500 steps of steepest descent, followed by 500 steps of conjugate gradient minimisation. The cut-off distance for non-bonded interactions was 10 Å and a force constant of 500 kcal/mol/Å^2^ was used to restrain the protein, while the ASP particles were held fixed. The entire system was then subjected to 2500 steps of minimisation without restraints, with the grids held fixed during the simulation using the belly function in AMBER. The central ASP particle was then activated (turned on) and the neighbouring ASP particles on the 3D grid were switched to a *latent* (receptive) status while all other particles remained inactive (turned off), see Figure 2a. MD simulations were performed at constant volume under periodic boundary condition. A 1 fs time step was used, and electrostatic interactions were represented using the smooth PME method. Before commencing the ASP simulations, the temperature was ramped from 0 to 300 K and weak atom restraints were reduced gradually from 100 kcal/mol/Å^2^ to 0 over a time course of 115 ps. The ASP simulations followed the protocol described by Withers *et al*. [30]. A new ASP particle was activated every 1 ps over a 400 ps run. System coordinates were saved every 1 ps for future analysis. Analyses of trajectories were performed with the *ptraj* module of AMBER and the PCAZIP suite of programs [78]. After re-centering the coordinates to the origin, re-imaging the trajectories to the primary boxes, and stripping of all water molecules and counter ions, the trajectories were converted into 400 snapshots and these were used to calculate the average coordinates for further analysis.

## 4. Conclusions

The aim of this paper was to investigate the dynamics of CDK5, GSK3β and ERK2 and to identify unique dynamical signatures of these proteins. To this end a number of homology models were built, including CDK5^D144N^/p25 in 5 different complexes, CDK5/p25, CDK5, GSK3β and ERK2. The structures were stable throughout the course of a 3.5 ns MD simulation. Model construction predicted the orientation of the flexible Gly-rich P-loop and other structurally variable regions in the structures that are missing in the X-ray crystal structures. The MD simulation of these proteins showed atomic fluctuations in the loop regions and could account for the unobserved features in the X-ray crystal structures. The dynamic studies of kinases showed conserved motions between the structures, yet ERK2 was significantly different from the other kinases studied. This precluded the need for further experiments to find distinguishable dynamics for ERK2. An examination of the atomic fluctuations of the CDK5 models showed them to be very similar, in agreement with the crystal structures, which justified the replacement of the wild-type residue Asp144 with the mutant Asn144 residue in these experiments and the need to examine the wild-type motion in the ASP experiments. The high level of similarity between the normal dynamics of apo and ligand bound mutant CDK5/p25 complexes necessitated further investigation using the ASP method.

ASP was then implemented in the study of CDK5/p25, CDK5 and GSK3β. A detailed examination of the dynamics using this method enabled distinction between the highly homologous kinase binding pockets and the identification of regions of conformational flexibility within the ATP-binding site.

A more elaborate analysis of the MD trajectories used PCA to provide a description of the conformational space occupied by the kinase systems. The Gly-rich P-loop, as predicted, is flexible, and this degree of flexibility can be used to distinguish between the kinases studied. The final stage of PCA examined the dynamic behaviour of each of the simulations in turn, and quantitatively compared the principal modes of motion exhibited by the protein backbone C_α_, C and N atoms.

It is thus important when designing receptor-based models to start with the optimal conformation of the receptor. The ASP protocol is able to induce conformational change within the ATP-binding site and generate a protein conformation that can account for the binding poses of small molecule inhibitors exemplified by the GSK3β example. The ASP method also opens up possibilities for further structure-based drug design methods. This method explores energetically feasible protein conformations, and suggest reasonable protein conformations which may be adopted upon ligand binding. These conformations could be suitable for creating an ensemble of structures for docking experiments. The ASP method demonstrates that the intrinsic flexibility within ligand-binding sites differs between GSK3β and CDK5, despite the high degree of homology between these proteins. The question remains as to whether this method could be exploited to design GSK3α/β isoform selective compounds identified in the literarture [79] given the high sequence similarity (97.5%) of these protein kinase domains.

In summary, there is considerable evidence from the MD simulations performed here that taking protein flexibility into account can help distinguish between otherwise highly homologous proteins. The results of the ASP simulation also yield protein conformations that can be of use in further structure-based design strategies.

## Supplementary Materials

Supplementary materials can be accessed at: http://www.mdpi.com/1420-3049/19/7/9134/s1.

## References

[1] Semple D., Smyth R., Burns J., Darjee R., McIntosh A. (2005). Oxford Handbook of Pyschiatry.

[2] Kuljis R.O. Alzheimer Disease. http://www.emedicine.com/neuro/topic13.htm/.

[3] Crowther R.A., Olesen O.F., Jakes R., Goedert M. (1992). The microtubule binding repeats of tau protein assemble into filaments like those found in Alzheimer’s disease. FEBS Lett..

[4] Goedert M., Spillantini M.G., Jakes R., Rutherford D., Crowther R.A. (1989). Multiple isoforms of human microtubule-associated protein tau: Sequences and localization in neurofibrillary tangles of Alzheimer’s disease. Neuron.

[5] Goedert M. (1993). Tau protein and the neurofibrillary pathology of Alzheimer’s disease. Trends Neurosci..

[6] Goedert M. (1996). Tau protein and the neurofibrillary pathology of Alzheimer’s disease. Ann. N. Y. Acad. Sci..

[7] Hardy J. (2003). The relationship between amyloid and tau. J. Mol. Neurosci..

[8] Mandelkow E., Song Y.H., Schweers O., Marx A., Mandelkow E.M. (1995). On the structure of microtubules, tau, and paired helical filaments. Neurobiol. Aging.

[9] Selkoe D.J. (1998). The cell biology of beta-amyloid precursor protein and presenilin in Alzheimer’s disease. Trends Cell. Biol..

[10] Johnson L.N., Noble M.E., Owen D.J. (1996). Active and inactive protein kinases: Structural basis for regulation. Cell.

[11] Mazanetz M.P., Fischer P.M. (2007). Untangling tau hyperphosphorylation in drug design for neurodegenerative diseases. Nat. Rev. Drug Discov..

[12] Shelton S.B., Johnson G.V. (2004). Cyclin-dependent kinase-5 in neurodegeneration. J. Neurochem..

[13] Spittaels K., van den Haute C., van Dorpe J., Geerts H., Mercken M., Bruynseels K., Lasrado R., Vandezande K., Laenen I., Boon T. (2000). Glycogen synthase kinase-3beta phosphorylates protein tau and rescues the axonopathy in the central nervous system of human four-repeat tau transgenic mice. J. Biol. Chem..

[14] Perry G., Roder H., Nunomura A., Takeda A., Friedlich A.L., Zhu X., Raina A.K., Holbrook N., Siedlak S.L., Harris P.L. (1999). Activation of neuronal extracellular receptor kinase (ERK) in Alzheimer disease links oxidative stress to abnormal phosphorylation. Neuroreport.

[15] Jeffrey P.D., Russo A.A., Polyak K., Gibbs E., Hurwitz J., Massague J., Pavletich N.P. (1995). Mechanism of CDK activation revealed by the structure of a cyclinA-CDK2 complex. Nature.

[16] Otyepka M., Bartova I., Kriz Z., Koca J. (2006). Different Mechanisms of CDK5 and CDK2 Activation as Revealed by CDK5/p25 and CDK2/Cyclin A Dynamics. J. Biol. Chem..

[17] Pande V., Ramos M.J. (2005). Structural basis for the GSK-3beta binding affinity and selectivity against CDK-2 of 1-(4-aminofurazan-3yl)-5-dialkylaminomethyl-1H-[1,2,3] triazole-4-carboxylic acid derivatives. Bioorg. Med. Chem. Lett..

[18] Rastelli G., Rosenfeld R., Reid R., Santi D.V. (2008). Molecular modeling and crystal structure of ERK2-hypothemycin complexes. J. Struct. Biol..

[19] Zhang B., Tan V.B.C., Lim K.M., Tay T.E. (2006). Molecular dynamics simulations on the inhibition of Cyclin-Dependent Kinases 2 and 5 in the presence of activators. J. Comput. Aided Mol. Des..

[20] Zhang B., Tan V.B., Lim K.M., Tay T.E., Zhuang S. (2007). Study of the inhibition of cyclin-dependent kinases with roscovitine and indirubin-3'-oxime from molecular dynamics simulations. J. Mol. Model..

[21] Zhang B., Tan V.B.C., Lim K.M., Tay T.E. (2007). Significance of Water Molecules in the Inhibition of Cylin-Dependent Kinase 2 and 5 Complexes. J. Chem. Inf. Model..

[22] De Azevedo W.F., Gaspar R.T., Canduri F., Camera J.C., da Silveira N.J.F. (2002). Molecular model of cyclin-dependent kinase 5 complexed with roscovitine. Biochem. Biophys. Res. Commun..

[23] Hilser V.J., Garcia-Moreno E.B., Oas T.G., Kapp G., Whitten S.T. (2006). A statistical thermodynamic model of the protein ensemble. Chem. Rev..

[24] Jacobs D.J., Rader A.J., Kuhn L.A., Thorpe M.F. (2001). Protein flexibility predictions using graph theory. Proteins.

[25] Ming L., Maria I.Z., Leslie A.K., Thorpe M.F. (2004). Sampling protein conformations and pathways. J. Comput. Chem..

[26] Seeliger D., Haas J., de Groot B.L. (2007). Geometry-based sampling of conformational transitions in proteins. Structure.

[27] Hayward S., de Groot B.L. (2008). Normal modes and essential dynamics. Methods Mol. Biol..

[28] Ahmed A., Gohlke H. (2006). Multiscale modeling of macromolecular conformational changes combining concepts from rigidity and elastic network theory. Proteins.

[29] Hegler J.A., Latzer J., Shehu A., Clementi C., Wolynes P.G. (2009). Restriction *versus* guidance in protein structure prediction. Proc. Natl. Acad. Sci. USA.

[30] Withers I.M., Mazanetz M.P., Wang H., Fischer P.M., Laughton C.A. (2008). Active Site Pressurization: A New Tool for Structure-Guided Drug Design and Other Studies of Protein Flexibility. J. Chem. Inf. Model..

[31] Mazanetz M.P., Withers I.M., Laughton C.A., Fischer P.M. (2008). Exploiting glycogen synthase kinase 3β flexibility in molecular recognition. Biochem. Soc. Trans..

[32] Mazanetz M.P., Withers I.M., Laughton C.A., Fischer P.M. (2009). A Study of CDK2 inhibitors using a novel 3D-QSAR method exploiting receptor flexibility. QSAR Comb. Sci..

[33] Case D.A., Pearlman D.A., Caldwell J.W., Cheatham T.E., Wang J., Ross W.S., Simmerling C.L., Darden T.A., Merz K.M., Stanton R.V. (2002). AMBER 7.

[34] (2009). The Molecular Operating Environment (MOE).

[35] DeLano W. (2006). The PyMOL Molecular Graphics System.

[36] Bertrand J.A., Thieffine S., Vulpetti A., Cristiani C., Valsasina B., Knapp S., Kalisz H.M., Flocco M. (2003). Structural characterization of the GSK-3beta active site using selective and non-selective ATP-mimetic inhibitors. J. Mol. Biol..

[37] Dajani R., Fraser E., Roe S.M., Young N., Good V., Dale T.C., Pearl L.H. (2001). Crystal structure of glycogen synthase kinase 3 beta: Structural basis for phosphate-primed substrate specificity and autoinhibition. Cell.

[38] Goldsmith E.J., Cobb M.H. (1994). Protein kinases. Curr. Opin. Struct. Biol..

[39] Amadei A., Linssen A.B., Berendsen H.J. (1993). Essential dynamics of proteins. Proteins.

[40] Amadei A., Ceruso M.A., di Nola A. (1999). On the convergence of the conformational coordinates basis set obtained by the essential dynamics analysis of proteins’ molecular dynamics simulations. Proteins.

[41] Garcia A.E. (1992). Large-amplitude nonlinear motions in proteins. Phys. Rev. Lett..

[42] Aschi M., Roccatano D., di Nola A., Gallina C., Gavuzzo E., Pochetti G., Pieper M., Tschesche H., Mazza F. (2002). Computational study of the catalytic domain of human neutrophil collagenase. Specific role of the S3 and S'3 subsites in the interaction with a phosphonate inhibitor. J. Comput. Aided Mol. Des..

[43] Hess B. (2000). Similarities between principal components of protein dynamics and random diffusion. Phys. Rev. E.

[44] Merlino A., Vitagliano L., Ceruso M.A., Mazzarella L. (2003). Subtle functional collective motions in pancreatic-like ribonucleases: From ribonuclease A to angiogenin. Proteins.

[45] Frisch M.J., Trucks G.W., Schlegel H.B., Scuseria G.E., Robb M.A., Cheeseman J.R., Zakrzewski V.G., Montgomery J.A., Stratmann R.E., Burant J.C. (2001). Gaussian 98.

[46] (1995). LEaP.

[47] Wang J., Wang W., Kollman P.A., Case D.A. (2006). Automatic atom type and bond type perception in molecular mechanical calculations. J. Mol. Graph. Modell..

[48] Wang J., Wolf R.M., Caldwell J.W., Kollman P.A., Case D.A. (2004). Development and testing of a general amber force field. J. Comput. Chem..

[49] Meagher K.L., Redman L.T., Carlson H.A. (2003). Development of polyphosphate parameters for use with the AMBER force field. J. Comput. Chem..

[50] Crowley M., Darden T., Cheatham T., Deerfield D. (1997). Adventures in improving the scaling and accuracy of a parallel molecular dynamics program. J. Supercomput..

[51] Pearlman D.A., Case D.A., Caldwell J.W., Ross W.S., Cheatham T.E., DeBolt S., Ferguson D., Seibel G., Kollman P. (1995). AMBER, a package of computer programs for applying molecular mechanics, normal mode analysis, molecular dynamics and free energy calculations to simulate the structural and energetic properties of molecules. Comput. Phys. Commun..

[52] Pettersen E.F., Goddard T.D., Huang C.C., Couch G.S., Greenblatt D.M., Meng E.C., Ferrin T.E. (2004). UCSF Chimera—A visualization system for exploratory research and analysis. J. Comput..

[53] Duan Y., Wu C., Chowdhury S., Lee M.C., Xiong G., Zhang W., Yang R., Cieplak P., Luo R., Lee T. (2003). A point-charge force field for molecular mechanics simulations of proteins based on condensed-phase quantum mechanical calculations. J. Comput. Chem..

[54] Jorgensen W.L. (1982). Revised TIPS for simulations of liquid water and aqueous solutions. J. Chem. Phys..

[55] Jorgensen W.L., Chandrasekhar J., Madura J.D., Impey R.W., Klein M.L. (1983). Comparison of simple potential functions for simulating liquid water. J. Chem. Phys..

[56] Darden T., York D., Pedersen L. (1998). Particle mesh Ewald—An Nlog(N) method for Ewald sums in large systems. J. Chem. Phys..

[57] Case D.A., Darden T.A., Cheatham T.E., Simmerling C.L., Wang J., Duke R.E., Luo R., Merz K.M., Wang B., Pearlman D.A. (2004). AMBER 8.0.

[58] Ryckaert J.P., Ciccotti G., Berendsen H.J.C. (1977). Numerical integration of the cartesian equations of motion of a system with constraints: Molecular dynmaics of n-alkanes. J. Comput. Phys..

[59] Berman H.M., Westbrook J., Feng Z., Gilliland G., Bhat T.N., Weissig H., Shindyalov I.N., Bourne P.E. (2000). The Protein Data Bank. Nucleic Acids Res..

[60] Pearson W.R., Lipman D.J. (1988). Improved tools for biological sequence comparison. Proc. Natl. Acad. Sci. USA.

[61] Altschul S.F., Gish W., Miller W., Myers E.W., Lipman D.J. (1990). Basic local alignment search tool. J. Mol. Biol..

[62] Altschul S.F., Madden T.L., Schaffer A.A., Zhang J., Zhang Z., Miller W., Lipman D.J. (1997). Gapped BLAST and PSI-BLAST: A new generation of protein database search programs. Nucleic Acids Res..

[63] Altschul S.F., Gish W., Miller W., Myers E.W., Lipman D.J. NCBI-BLAST. http://blast.ncbi.nlm.nih.gov/Blast.cgi/.

[64] Henikoff S., Henikoff J.G. (1992). Amino acid substitution matrices from protein blocks. Proc. Natl. Acad. Sci. USA.

[65] Meng E., Pettersen E.F., Couch G.S., Huang C.C., Ferrin T.E. (2006). Tools for integrated sequence-structure analysis with UCSF Chimera. BMC Bioinform..

[66] Needleman S.B., Wunsch C.D. (1970). A general method applicable to the search for similarities in the amino acid sequence of two proteins. J. Mol. Biol..

[67] Thompson J.D., Higgins D.G., Gibson T.J. (1994). CLUSTAL W: Improving the sensitivity of progressive multiple sequence alignment through sequence weighting, position-specific gap penalties and weight matrix choice. Nucleic Acids Res..

[68] SDSC Biology Workbench. http://seqtool.sdsc.edu/.

[69] Sali A., Blundell T.L. (1993). Comparative protein modelling by satisfaction of spatial restraints. J. Mol. Biol..

[70] Mapelli M., Massimiliano L., Crovace C., Seeliger M.A., Tsai L.H., Meijer L., Musacchio A. (2005). Mechanism of CDK5/p25 binding by CDK inhibitors. J. Med. Chem..

[71] Tarricone C., Dhavan R., Peng J., Areces L.B., Tsai L.H., Musacchio A. (2001). Structure and regulation of the CDK5-p25(NCK5a) complex. Mol. Cell..

[72] Russo A.A., Jeffrey P.D., Pavletich N.P. (1996). Structural basis of cyclin-dependent kinase activation by phosphorylation. Nat Struct. Biol..

[73] Wu S.Y., McNae I., Kontopidis G., McClue S.J., McInnes C., Stewart K.J., Wang S., Zheleva D.I., Marriage H., Lane D.P. (2003). Walkinshaw, M.D. Discovery of a novel family of CDK inhibitors with the program LIDAEUS: Structural basis for ligand-induced disordering of the activation loop. Structure.

[74] Ter Haar E., Coll J.T., Austen D.A., Hsiao H.M., Swenson L., Jain J. (2001). Structure of GSK3beta reveals a primed phosphorylation mechanism. Nat. Struct. Biol..

[75] Bhat R., Xue Y., Berg S., Hellberg S., Ormo M., Nilsson Y., Radesater A.C., Jerning E., Markgren P.O., Borgegard T. (2003). Structural insights and biological effects of glycogen synthase kinase 3-specific inhibitor AR-A014418. J. Biol. Chem..

[76] Ohori M., Kinoshita T., Okubo M., Sato K., Yamazaki A., Arakawa H., Nishimura S., Inamura N., Nakajima H., Neya M. (2005). Identification of a selective ERK inhibitor and structural determination of the inhibitor-ERK2 complex. Biochem. Biophys. Res. Commun..

[77] (2006). SYBYL.

[78] Meyer T., Ferrer-Costa C., Perez A., Rueda M., Bidon-Chanal A., Luque F.J., Laughton C.A., Orozco M. (2006). Essential Dynamics: A Tool for Efficient Trajectory Compression and Management. J. Chem. Theory Comput..

[79] Karaman M.W., Herrgard S., Treiber D.K., Gallant P., Atteridge C.E., Campbell B.T., Chan K.W., Ciceri P., Davis M.I., Edeen P.T. (2008). A quantitative analysis of kinase inhibitor selectivity. Nat. Biotech..

