# Supplementary material for: Investigation of the Flexibility of Protein Kinases Implicated in the Pathology of Alzheimer’s Disease
Source: Molecules. 2014 Jun 30;19(7):9134–59. doi: 10.3390/molecules19079134 (PMC6270768; doi:10.3390/molecules19079134)
Supplement: Supplementary file 1 [file molecules-19-09134-s001.zip › molecules-56295-supplementary-final/Supporting Information.docx]

Supporting Information

Contents: AMBER parameter files for *R*-roscovitine (roscov.lib), aloisine-A (aloisine.lib), indirubin-3'-oxime (indirubin.lib) and ATP (ATP.lib).

**roscov.lib**

!!index array str

"MOL"

!entry.MOL.unit.atoms table str name str type int typex int resx int flags int seq int elmnt dbl chg

"O1" "oh" 0 1 131072 1 8 -0.479400

"H13" "ho" 0 1 131072 2 1 0.302700

"C16" "c3" 0 1 131072 3 6 0.147600

"H14" "h1" 0 1 131072 4 1 0.013800

"H15" "h1" 0 1 131072 5 1 0.013800

"C15" "c3" 0 1 131072 6 6 0.033100

"C17" "c3" 0 1 131072 7 6 -0.236400

"C18" "c3" 0 1 131072 8 6 -0.080900

"H16" "hc" 0 1 131072 9 1 0.028700

"H17" "hc" 0 1 131072 10 1 0.028700

"H18" "hc" 0 1 131072 11 1 0.028700

"H19" "hc" 0 1 131072 12 1 0.078100

"H20" "hc" 0 1 131072 13 1 0.078100

"H21" "h1" 0 1 131072 14 1 0.098900

"N6" "nh" 0 1 131072 15 7 -0.403300

"H22" "hn" 0 1 131072 16 1 0.201200

"C11" "ca" 0 1 131072 17 6 0.515800

"N3" "nb" 0 1 131072 18 7 -0.479800

"N2" "nb" 0 1 131072 19 7 -0.216100

"C10" "ca" 0 1 131072 20 6 0.029800

"N5" "na" 0 1 131072 21 7 0.019200

"C12" "cd" 0 1 131072 22 6 0.180300

"N4" "nc" 0 1 131072 23 7 -0.540500

"H4" "h5" 0 1 131072 24 1 0.064200

"C13" "c3" 0 1 131072 25 6 0.008100

"C14" "c3" 0 1 131072 26 6 -0.098600

"H1" "hc" 0 1 131072 27 1 0.034300

"H2" "hc" 0 1 131072 28 1 0.034300

"H3" "hc" 0 1 131072 29 1 0.034300

"C19" "c3" 0 1 131072 30 6 -0.098600

"H23" "hc" 0 1 131072 31 1 0.034300

"H24" "hc" 0 1 131072 32 1 0.034300

"H25" "hc" 0 1 131072 33 1 0.034300

"H26" "h1" 0 1 131072 34 1 0.047300

"C9" "ca" 0 1 131072 35 6 0.227500

"C8" "ca" 0 1 131072 36 6 0.223900

"N1" "nh" 0 1 131072 37 7 -0.213800

"H12" "hn" 0 1 131072 38 1 0.187100

"C7" "c3" 0 1 131072 39 6 -0.033300

"H10" "h1" 0 1 131072 40 1 0.045800

"H11" "h1" 0 1 131072 41 1 0.045800

"C2" "ca" 0 1 131072 42 6 0.075300

"C1" "ca" 0 1 131072 43 6 -0.064700

"H5" "ha" 0 1 131072 44 1 0.053700

"C6" "ca" 0 1 131072 45 6 -0.087700

"H6" "ha" 0 1 131072 46 1 0.069300

"C5" "ca" 0 1 131072 47 6 -0.051500

"H7" "ha" 0 1 131072 48 1 0.062000

"C4" "ca" 0 1 131072 49 6 -0.087700

"H8" "ha" 0 1 131072 50 1 0.069300

"C3" "ca" 0 1 131072 51 6 -0.064700

"H9" "ha" 0 1 131072 52 1 0.053700

!entry.MOL.unit.atomspertinfo table str pname str ptype int ptypex int pelmnt dbl pchg

"O1" "oh" 0 -1 0.0

"H13" "ho" 0 -1 0.0

"C16" "c3" 0 -1 0.0

"H14" "h1" 0 -1 0.0

"H15" "h1" 0 -1 0.0

"C15" "c3" 0 -1 0.0

"C17" "c3" 0 -1 0.0

"C18" "c3" 0 -1 0.0

"H16" "hc" 0 -1 0.0

"H17" "hc" 0 -1 0.0

"H18" "hc" 0 -1 0.0

"H19" "hc" 0 -1 0.0

"H20" "hc" 0 -1 0.0

"H21" "h1" 0 -1 0.0

"N6" "nh" 0 -1 0.0

"H22" "hn" 0 -1 0.0

"C11" "ca" 0 -1 0.0

"N3" "nb" 0 -1 0.0

"N2" "nb" 0 -1 0.0

"C10" "ca" 0 -1 0.0

"N5" "na" 0 -1 0.0

"C12" "cd" 0 -1 0.0

"N4" "nc" 0 -1 0.0

"H4" "h5" 0 -1 0.0

"C13" "c3" 0 -1 0.0

"C14" "c3" 0 -1 0.0

"H1" "hc" 0 -1 0.0

"H2" "hc" 0 -1 0.0

"H3" "hc" 0 -1 0.0

"C19" "c3" 0 -1 0.0

"H23" "hc" 0 -1 0.0

"H24" "hc" 0 -1 0.0

"H25" "hc" 0 -1 0.0

"H26" "h1" 0 -1 0.0

"C9" "ca" 0 -1 0.0

"C8" "ca" 0 -1 0.0

"N1" "nh" 0 -1 0.0

"H12" "hn" 0 -1 0.0

"C7" "c3" 0 -1 0.0

"H10" "h1" 0 -1 0.0

"H11" "h1" 0 -1 0.0

"C2" "ca" 0 -1 0.0

"C1" "ca" 0 -1 0.0

"H5" "ha" 0 -1 0.0

"C6" "ca" 0 -1 0.0

"H6" "ha" 0 -1 0.0

"C5" "ca" 0 -1 0.0

"H7" "ha" 0 -1 0.0

"C4" "ca" 0 -1 0.0

"H8" "ha" 0 -1 0.0

"C3" "ca" 0 -1 0.0

"H9" "ha" 0 -1 0.0

!entry.MOL.unit.boundbox array dbl

-1.000000

0.0

0.0

0.0

0.0

!entry.MOL.unit.childsequence single int

2

!entry.MOL.unit.connect array int

1

51

!entry.MOL.unit.connectivity table int atom1x int atom2x int flags

1 2 1

1 3 1

3 4 1

3 5 1

3 6 1

6 7 1

6 14 1

6 15 1

7 8 1

7 12 1

7 13 1

8 9 1

8 10 1

8 11 1

15 16 1

15 17 1

17 18 1

17 19 1

18 36 1

19 20 1

20 21 1

20 35 1

21 22 1

21 25 1

22 23 1

22 24 1

23 35 1

25 26 1

25 30 1

25 34 1

26 27 1

26 28 1

26 29 1

30 31 1

30 32 1

30 33 1

35 36 1

36 37 1

37 38 1

37 39 1

39 40 1

39 41 1

39 42 1

42 43 1

42 51 1

43 44 1

43 45 1

45 46 1

45 47 1

47 48 1

47 49 1

49 50 1

49 51 1

51 52 1

!entry.MOL.unit.hierarchy table str abovetype int abovex str belowtype int belowx

"U" 0 "R" 1

"R" 1 "A" 1

"R" 1 "A" 2

"R" 1 "A" 3

"R" 1 "A" 4

"R" 1 "A" 5

"R" 1 "A" 6

"R" 1 "A" 7

"R" 1 "A" 8

"R" 1 "A" 9

"R" 1 "A" 10

"R" 1 "A" 11

"R" 1 "A" 12

"R" 1 "A" 13

"R" 1 "A" 14

"R" 1 "A" 15

"R" 1 "A" 16

"R" 1 "A" 17

"R" 1 "A" 18

"R" 1 "A" 19

"R" 1 "A" 20

"R" 1 "A" 21

"R" 1 "A" 22

"R" 1 "A" 23

"R" 1 "A" 24

"R" 1 "A" 25

"R" 1 "A" 26

"R" 1 "A" 27

"R" 1 "A" 28

"R" 1 "A" 29

"R" 1 "A" 30

"R" 1 "A" 31

"R" 1 "A" 32

"R" 1 "A" 33

"R" 1 "A" 34

"R" 1 "A" 35

"R" 1 "A" 36

"R" 1 "A" 37

"R" 1 "A" 38

"R" 1 "A" 39

"R" 1 "A" 40

"R" 1 "A" 41

"R" 1 "A" 42

"R" 1 "A" 43

"R" 1 "A" 44

"R" 1 "A" 45

"R" 1 "A" 46

"R" 1 "A" 47

"R" 1 "A" 48

"R" 1 "A" 49

"R" 1 "A" 50

"R" 1 "A" 51

"R" 1 "A" 52

!entry.MOL.unit.name single str

"MOL"

!entry.MOL.unit.positions table dbl x dbl y dbl z

3.536914 1.422858 -1.933042E-06

3.697674 0.495087 0.110006

4.331295 2.145130 0.904706

5.373028 1.836055 0.832698

4.269336 3.179136 0.602658

3.853510 1.990205 2.350971

4.836203 2.619983 3.344843

4.410410 2.475161 4.804108

4.255306 1.431421 5.069237

3.488680 3.007267 4.999048

5.175566 2.871550 5.465023

4.976214 3.666162 3.106870

5.797493 2.130609 3.202788

3.832522 0.922859 2.566038

2.470770 2.406583 2.528973

1.790405 1.697598 2.379042

1.952255 3.647000 2.310914

0.611139 3.652476 2.208922

2.766066 4.684251 2.245935

2.109954 5.832883 2.065968

2.619620 7.095012 1.950955

1.529321 7.914747 1.778014

0.405760 7.305766 1.772024

1.657246 8.972532 1.660078

4.011373 7.540542 2.020916

4.600921 7.283632 3.406994

4.637959 6.222437 3.620026

4.003919 7.766555 4.172128

5.610026 7.681497 3.459977

4.848395 6.928250 0.900103

5.850355 7.344065 0.920094

4.408900 7.144447 -0.068054

4.918449 5.854345 1.012104

3.966328 8.612174 1.863969

0.749630 5.982303 1.954967

-0.012035 4.801014 2.037939

-1.352198 4.833473 1.956939

-1.755807 5.705588 1.698987

-2.180881 3.645973 1.881922

-1.827561 2.943665 2.623872

-2.074958 3.163200 0.914912

-3.633149 3.991095 2.128857

-4.596433 3.749330 1.162094

-4.307537 3.321813 0.217878

-5.931119 4.053801 1.397109

-6.664416 3.859487 0.635438

-6.312149 4.608982 2.603970

-7.344228 4.846913 2.787889

-5.354854 4.857736 3.577752

-5.644560 5.287974 4.519342

-4.028102 4.551237 3.341719

-3.289731 4.745568 4.098467

!entry.MOL.unit.residueconnect table int c1x int c2x int c3x int c4x int c5x int c6x

1 51 0 0 0 0

!entry.MOL.unit.residues table str name int seq int childseq int startatomx str restype int imagingx

"MOL" 1 53 1 "?" 0

!entry.MOL.unit.residuesPdbSequenceNumber array int

0

!entry.MOL.unit.solventcap array dbl

-1.000000

0.0

0.0

0.0

0.0

!entry.MOL.unit.velocities table dbl x dbl y dbl z

0.0 0.0 0.0

0.0 0.0 0.0

0.0 0.0 0.0

0.0 0.0 0.0

0.0 0.0 0.0

0.0 0.0 0.0

0.0 0.0 0.0

0.0 0.0 0.0

0.0 0.0 0.0

0.0 0.0 0.0

0.0 0.0 0.0

0.0 0.0 0.0

0.0 0.0 0.0

0.0 0.0 0.0

0.0 0.0 0.0

0.0 0.0 0.0

0.0 0.0 0.0

0.0 0.0 0.0

0.0 0.0 0.0

0.0 0.0 0.0

0.0 0.0 0.0

0.0 0.0 0.0

0.0 0.0 0.0

0.0 0.0 0.0

0.0 0.0 0.0

0.0 0.0 0.0

0.0 0.0 0.0

0.0 0.0 0.0

0.0 0.0 0.0

0.0 0.0 0.0

0.0 0.0 0.0

0.0 0.0 0.0

0.0 0.0 0.0

0.0 0.0 0.0

0.0 0.0 0.0

0.0 0.0 0.0

0.0 0.0 0.0

0.0 0.0 0.0

0.0 0.0 0.0

0.0 0.0 0.0

0.0 0.0 0.0

0.0 0.0 0.0

0.0 0.0 0.0

0.0 0.0 0.0

0.0 0.0 0.0

0.0 0.0 0.0

0.0 0.0 0.0

0.0 0.0 0.0

0.0 0.0 0.0

0.0 0.0 0.0

0.0 0.0 0.0

0.0 0.0 0.0

**aloisine.lib**

!!index array str

"MOL"

!entry.MOL.unit.atoms table str name str type int typex int resx int flags int seq int elmnt dbl chg

"C12" "ca" 0 1 131072 1 6 0.076000

"N3" "nb" 0 1 131072 2 7 -0.386300

"H1" "h4" 0 1 131072 3 1 0.019000

"C11" "ca" 0 1 131072 4 6 0.249800

"H17" "h4" 0 1 131072 5 1 -0.001700

"N2" "nb" 0 1 131072 6 7 -0.550400

"C10" "ca" 0 1 131072 7 6 0.522400

"N1" "na" 0 1 131072 8 7 -0.506100

"H16" "hn" 0 1 131072 9 1 0.309200

"C9" "ca" 0 1 131072 10 6 0.236600

"C8" "cd" 0 1 131072 11 6 -0.109200

"C13" "c3" 0 1 131072 12 6 -0.193700

"C14" "c3" 0 1 131072 13 6 -0.046800

"C15" "c3" 0 1 131072 14 6 -0.019200

"C16" "c3" 0 1 131072 15 6 -0.101800

"H8" "hc" 0 1 131072 16 1 0.027200

"H9" "hc" 0 1 131072 17 1 0.027200

"H10" "hc" 0 1 131072 18 1 0.027200

"H6" "hc" 0 1 131072 19 1 0.022100

"H7" "hc" 0 1 131072 20 1 0.022100

"H4" "hc" 0 1 131072 21 1 0.037600

"H5" "hc" 0 1 131072 22 1 0.037600

"H2" "hc" 0 1 131072 23 1 0.078100

"H3" "hc" 0 1 131072 24 1 0.078100

"C7" "cc" 0 1 131072 25 6 0.057000

"C2" "ca" 0 1 131072 26 6 0.118300

"C1" "ca" 0 1 131072 27 6 -0.106200

"H11" "ha" 0 1 131072 28 1 0.080000

"C6" "ca" 0 1 131072 29 6 -0.163200

"H12" "ha" 0 1 131072 30 1 0.089000

"C5" "ca" 0 1 131072 31 6 0.317200

"O1" "oh" 0 1 131072 32 8 -0.462200

"H13" "ho" 0 1 131072 33 1 0.316000

"C4" "ca" 0 1 131072 34 6 -0.163200

"H14" "ha" 0 1 131072 35 1 0.089000

"C3" "ca" 0 1 131072 36 6 -0.106200

"H15" "ha" 0 1 131072 37 1 0.080000

!entry.MOL.unit.atomspertinfo table str pname str ptype int ptypex int pelmnt dbl pchg

"C12" "ca" 0 -1 0.0

"N3" "nb" 0 -1 0.0

"H1" "h4" 0 -1 0.0

"C11" "ca" 0 -1 0.0

"H17" "h4" 0 -1 0.0

"N2" "nb" 0 -1 0.0

"C10" "ca" 0 -1 0.0

"N1" "na" 0 -1 0.0

"H16" "hn" 0 -1 0.0

"C9" "ca" 0 -1 0.0

"C8" "cd" 0 -1 0.0

"C13" "c3" 0 -1 0.0

"C14" "c3" 0 -1 0.0

"C15" "c3" 0 -1 0.0

"C16" "c3" 0 -1 0.0

"H8" "hc" 0 -1 0.0

"H9" "hc" 0 -1 0.0

"H10" "hc" 0 -1 0.0

"H6" "hc" 0 -1 0.0

"H7" "hc" 0 -1 0.0

"H4" "hc" 0 -1 0.0

"H5" "hc" 0 -1 0.0

"H2" "hc" 0 -1 0.0

"H3" "hc" 0 -1 0.0

"C7" "cc" 0 -1 0.0

"C2" "ca" 0 -1 0.0

"C1" "ca" 0 -1 0.0

"H11" "ha" 0 -1 0.0

"C6" "ca" 0 -1 0.0

"H12" "ha" 0 -1 0.0

"C5" "ca" 0 -1 0.0

"O1" "oh" 0 -1 0.0

"H13" "ho" 0 -1 0.0

"C4" "ca" 0 -1 0.0

"H14" "ha" 0 -1 0.0

"C3" "ca" 0 -1 0.0

"H15" "ha" 0 -1 0.0

!entry.MOL.unit.boundbox array dbl

-1.000000

0.0

0.0

0.0

0.0

!entry.MOL.unit.childsequence single int

2

!entry.MOL.unit.connect array int

1

36

!entry.MOL.unit.connectivity table int atom1x int atom2x int flags

1 2 1

1 3 1

1 4 1

2 10 1

4 5 1

4 6 1

6 7 1

7 8 1

7 10 1

8 9 1

8 25 1

10 11 1

11 12 1

11 25 1

12 13 1

12 23 1

12 24 1

13 14 1

13 21 1

13 22 1

14 15 1

14 19 1

14 20 1

15 16 1

15 17 1

15 18 1

25 26 1

26 27 1

26 36 1

27 28 1

27 29 1

29 30 1

29 31 1

31 32 1

31 34 1

32 33 1

34 35 1

34 36 1

36 37 1

!entry.MOL.unit.hierarchy table str abovetype int abovex str belowtype int belowx

"U" 0 "R" 1

"R" 1 "A" 1

"R" 1 "A" 2

"R" 1 "A" 3

"R" 1 "A" 4

"R" 1 "A" 5

"R" 1 "A" 6

"R" 1 "A" 7

"R" 1 "A" 8

"R" 1 "A" 9

"R" 1 "A" 10

"R" 1 "A" 11

"R" 1 "A" 12

"R" 1 "A" 13

"R" 1 "A" 14

"R" 1 "A" 15

"R" 1 "A" 16

"R" 1 "A" 17

"R" 1 "A" 18

"R" 1 "A" 19

"R" 1 "A" 20

"R" 1 "A" 21

"R" 1 "A" 22

"R" 1 "A" 23

"R" 1 "A" 24

"R" 1 "A" 25

"R" 1 "A" 26

"R" 1 "A" 27

"R" 1 "A" 28

"R" 1 "A" 29

"R" 1 "A" 30

"R" 1 "A" 31

"R" 1 "A" 32

"R" 1 "A" 33

"R" 1 "A" 34

"R" 1 "A" 35

"R" 1 "A" 36

"R" 1 "A" 37

!entry.MOL.unit.name single str

"MOL"

!entry.MOL.unit.positions table dbl x dbl y dbl z

3.536914 1.422858 -1.933042E-06

2.660058 2.383240 0.232010

4.574555 1.655826 0.157004

3.166300 0.150107 -0.428020

3.915672 -0.601443 -0.599014

1.907951 -0.197264 -0.643013

1.052077 0.765205 -0.414980

-0.300813 0.728660 -0.548966

-0.833397 -0.102561 -0.664996

1.398744 2.054137 0.030132

0.176718 2.806601 0.190088

0.098992 4.257598 0.578137

0.637352 5.201860 -0.507143

0.574587 6.672867 -0.091091

1.104881 7.618016 -1.168248

0.531181 7.526980 -2.087024

1.050948 8.652745 -0.846295

2.141859 7.399052 -1.405159

-0.452941 6.938945 0.147935

1.147127 6.811892 0.823575

1.663004 4.933752 -0.738186

0.061741 5.056807 -1.418941

-0.928164 4.523587 0.800076

0.666959 4.408716 1.492547

-0.822679 1.952735 -0.150914

-2.290326 2.141022 -0.181916

-3.017645 1.826718 -1.322767

-2.506986 1.469684 -2.198769

-4.393114 1.984164 -1.361770

-4.936436 1.741254 -2.259391

-5.063822 2.461758 -0.248091

-6.401015 2.638229 -0.224107

-6.788354 2.399636 -1.054679

-4.355225 2.776172 0.903246

-4.888795 3.136081 1.763040

-2.986225 2.612789 0.931254

-2.447458 2.838892 1.833603

!entry.MOL.unit.residueconnect table int c1x int c2x int c3x int c4x int c5x int c6x

1 36 0 0 0 0

!entry.MOL.unit.residues table str name int seq int childseq int startatomx str restype int imagingx

"MOL" 1 38 1 "?" 0

!entry.MOL.unit.residuesPdbSequenceNumber array int

0

!entry.MOL.unit.solventcap array dbl

-1.000000

0.0

0.0

0.0

0.0

!entry.MOL.unit.velocities table dbl x dbl y dbl z

0.0 0.0 0.0

0.0 0.0 0.0

0.0 0.0 0.0

0.0 0.0 0.0

0.0 0.0 0.0

0.0 0.0 0.0

0.0 0.0 0.0

0.0 0.0 0.0

0.0 0.0 0.0

0.0 0.0 0.0

0.0 0.0 0.0

0.0 0.0 0.0

0.0 0.0 0.0

0.0 0.0 0.0

0.0 0.0 0.0

0.0 0.0 0.0

0.0 0.0 0.0

0.0 0.0 0.0

0.0 0.0 0.0

0.0 0.0 0.0

0.0 0.0 0.0

0.0 0.0 0.0

0.0 0.0 0.0

0.0 0.0 0.0

0.0 0.0 0.0

0.0 0.0 0.0

0.0 0.0 0.0

0.0 0.0 0.0

0.0 0.0 0.0

0.0 0.0 0.0

0.0 0.0 0.0

0.0 0.0 0.0

0.0 0.0 0.0

0.0 0.0 0.0

0.0 0.0 0.0

0.0 0.0 0.0

0.0 0.0 0.0

**indirubin.lib**

!!index array str

"MOL"

!entry.MOL.unit.atoms table str name str type int typex int resx int flags int seq int elmnt dbl chg

"O2" "oh" 0 1 131072 1 8 -0.332400

"H1" "ho" 0 1 131072 2 1 0.315700

"N3" "n2" 0 1 131072 3 7 -0.044900

"C7" "cc" 0 1 131072 4 6 0.166900

"C2" "ca" 0 1 131072 5 6 0.092600

"C1" "ca" 0 1 131072 6 6 -0.059700

"H6" "ha" 0 1 131072 7 1 0.056600

"C6" "ca" 0 1 131072 8 6 -0.101900

"H5" "ha" 0 1 131072 9 1 0.073900

"C5" "ca" 0 1 131072 10 6 -0.069000

"H4" "ha" 0 1 131072 11 1 0.074500

"C4" "ca" 0 1 131072 12 6 -0.087800

"H3" "ha" 0 1 131072 13 1 0.077200

"C3" "ca" 0 1 131072 14 6 -0.012400

"N1" "na" 0 1 131072 15 7 -0.131300

"H2" "hn" 0 1 131072 16 1 0.220800

"C8" "cc" 0 1 131072 17 6 -0.076100

"C9" "cd" 0 1 131072 18 6 -0.033500

"C12" "c" 0 1 131072 19 6 0.446000

"N2" "n" 0 1 131072 20 7 -0.326500

"H7" "hn" 0 1 131072 21 1 0.240300

"O1" "o" 0 1 131072 22 8 -0.416400

"C10" "ca" 0 1 131072 23 6 -0.029900

"C11" "ca" 0 1 131072 24 6 0.049000

"C13" "ca" 0 1 131072 25 6 -0.073900

"H8" "ha" 0 1 131072 26 1 0.069500

"C14" "ca" 0 1 131072 27 6 -0.113200

"H9" "ha" 0 1 131072 28 1 0.073800

"C15" "ca" 0 1 131072 29 6 -0.054900

"H10" "ha" 0 1 131072 30 1 0.061700

"C16" "ca" 0 1 131072 31 6 -0.053000

"H11" "ha" 0 1 131072 32 1 -0.001700

!entry.MOL.unit.atomspertinfo table str pname str ptype int ptypex int pelmnt dbl pchg

"O2" "oh" 0 -1 0.0

"H1" "ho" 0 -1 0.0

"N3" "n2" 0 -1 0.0

"C7" "cc" 0 -1 0.0

"C2" "ca" 0 -1 0.0

"C1" "ca" 0 -1 0.0

"H6" "ha" 0 -1 0.0

"C6" "ca" 0 -1 0.0

"H5" "ha" 0 -1 0.0

"C5" "ca" 0 -1 0.0

"H4" "ha" 0 -1 0.0

"C4" "ca" 0 -1 0.0

"H3" "ha" 0 -1 0.0

"C3" "ca" 0 -1 0.0

"N1" "na" 0 -1 0.0

"H2" "hn" 0 -1 0.0

"C8" "cc" 0 -1 0.0

"C9" "cd" 0 -1 0.0

"C12" "c" 0 -1 0.0

"N2" "n" 0 -1 0.0

"H7" "hn" 0 -1 0.0

"O1" "o" 0 -1 0.0

"C10" "ca" 0 -1 0.0

"C11" "ca" 0 -1 0.0

"C13" "ca" 0 -1 0.0

"H8" "ha" 0 -1 0.0

"C14" "ca" 0 -1 0.0

"H9" "ha" 0 -1 0.0

"C15" "ca" 0 -1 0.0

"H10" "ha" 0 -1 0.0

"C16" "ca" 0 -1 0.0

"H11" "ha" 0 -1 0.0

!entry.MOL.unit.boundbox array dbl

-1.000000

0.0

0.0

0.0

0.0

!entry.MOL.unit.childsequence single int

2

!entry.MOL.unit.connect array int

1

31

!entry.MOL.unit.connectivity table int atom1x int atom2x int flags

1 2 1

1 3 1

3 4 1

4 5 1

4 17 1

5 6 1

5 14 1

6 7 1

6 8 1

8 9 1

8 10 1

10 11 1

10 12 1

12 13 1

12 14 1

14 15 1

15 16 1

15 17 1

17 18 1

18 19 1

18 23 1

19 20 1

19 22 1

20 21 1

20 24 1

23 24 1

23 31 1

24 25 1

25 26 1

25 27 1

27 28 1

27 29 1

29 30 1

29 31 1

31 32 1

!entry.MOL.unit.hierarchy table str abovetype int abovex str belowtype int belowx

"U" 0 "R" 1

"R" 1 "A" 1

"R" 1 "A" 2

"R" 1 "A" 3

"R" 1 "A" 4

"R" 1 "A" 5

"R" 1 "A" 6

"R" 1 "A" 7

"R" 1 "A" 8

"R" 1 "A" 9

"R" 1 "A" 10

"R" 1 "A" 11

"R" 1 "A" 12

"R" 1 "A" 13

"R" 1 "A" 14

"R" 1 "A" 15

"R" 1 "A" 16

"R" 1 "A" 17

"R" 1 "A" 18

"R" 1 "A" 19

"R" 1 "A" 20

"R" 1 "A" 21

"R" 1 "A" 22

"R" 1 "A" 23

"R" 1 "A" 24

"R" 1 "A" 25

"R" 1 "A" 26

"R" 1 "A" 27

"R" 1 "A" 28

"R" 1 "A" 29

"R" 1 "A" 30

"R" 1 "A" 31

"R" 1 "A" 32

!entry.MOL.unit.name single str

"MOL"

!entry.MOL.unit.positions table dbl x dbl y dbl z

3.536914 1.422858 -1.933042E-06

4.072456 2.203885 -3.277374E-06

4.429757 0.395652 -3.552260E-06

3.875751 -0.732677 -3.403019E-06

2.456607 -1.127339 -1.811532E-06

1.266160 -0.417527 2.747385E-08

1.269143 0.652469 5.417773E-07

0.071759 -1.122633 -4.526389E-07

-0.862245 -0.590410 -3.066566E-07

0.076392 -2.511625 -2.733543E-06

-0.860017 -3.041639 -4.335736E-06

1.258916 -3.236474 -4.592173E-06

1.256950 -4.310472 -7.586919E-06

2.440754 -2.520107 -4.086503E-06

3.733597 -3.005615 -7.076387E-06

4.006290 -3.965637 -1.010121E-05

4.669730 -2.010823 -7.179821E-06

6.002127 -2.257286 -1.140628E-05

6.469943 -3.669833 -0.001017

7.829307 -3.628253 -2.801466E-05

8.382006 -4.454424 0.000960

5.817934 -4.687951 -2.608943E-05

7.224965 -1.430700 -1.409560E-05

8.308996 -2.322939 -2.012851E-05

9.621671 -1.910313 -2.563342E-05

10.427309 -2.622052 0.000965

9.870124 -0.541681 -2.511909E-05

10.886587 -0.188725 -3.060315E-05

8.824163 0.361585 -1.924694E-05

9.029154 1.416859 -2.016749E-05

7.500684 -0.072986 -1.371908E-05

6.706405 0.639481 -0.001009

!entry.MOL.unit.residueconnect table int c1x int c2x int c3x int c4x int c5x int c6x

1 31 0 0 0 0

!entry.MOL.unit.residues table str name int seq int childseq int startatomx str restype int imagingx

"MOL" 1 33 1 "?" 0

!entry.MOL.unit.residuesPdbSequenceNumber array int

0

!entry.MOL.unit.solventcap array dbl

-1.000000

0.0

0.0

0.0

0.0

!entry.MOL.unit.velocities table dbl x dbl y dbl z

0.0 0.0 0.0

0.0 0.0 0.0

0.0 0.0 0.0

0.0 0.0 0.0

0.0 0.0 0.0

0.0 0.0 0.0

0.0 0.0 0.0

0.0 0.0 0.0

0.0 0.0 0.0

0.0 0.0 0.0

0.0 0.0 0.0

0.0 0.0 0.0

0.0 0.0 0.0

0.0 0.0 0.0

0.0 0.0 0.0

0.0 0.0 0.0

0.0 0.0 0.0

0.0 0.0 0.0

0.0 0.0 0.0

0.0 0.0 0.0

0.0 0.0 0.0

0.0 0.0 0.0

0.0 0.0 0.0

0.0 0.0 0.0

0.0 0.0 0.0

0.0 0.0 0.0

0.0 0.0 0.0

0.0 0.0 0.0

0.0 0.0 0.0

0.0 0.0 0.0

0.0 0.0 0.0

0.0 0.0 0.0

**ATP.lib**

**!!index array str**

**"ATP"**

**!entry.ATP.unit.atoms table str name str type int typex int resx int flags int seq int elmnt dbl chg**

**"O1G" "O3" 0 1 131072 1 -1 -0.952600**

**"PG" "P" 0 1 131072 2 15 1.265000**

**"O2G" "O3" 0 1 131072 3 -1 -0.952600**

**"O3G" "O3" 0 1 131072 4 -1 -0.952600**

**"O3B" "OS" 0 1 131072 5 8 -0.532200**

**"PB" "P" 0 1 131072 6 15 1.385200**

**"O1B" "O2" 0 1 131072 7 8 -0.889400**

**"O2B" "O2" 0 1 131072 8 8 -0.889400**

**"O3A" "OS" 0 1 131072 9 8 -0.568900**

**"PA" "P" 0 1 131072 10 15 1.253200**

**"O1A" "O2" 0 1 131072 11 8 -0.879900**

**"O2A" "O2" 0 1 131072 12 8 -0.879900**

**"O5*" "OS" 0 1 131072 13 8 -0.598700**

**"C5*" "CT" 0 1 131072 14 6 0.055800**

**"H50" "H1" 0 1 131072 15 1 0.067900**

**"H51" "H1" 0 1 131072 16 1 0.067900**

**"C4*" "CT" 0 1 131072 17 6 0.106500**

**"H40" "H1" 0 1 131072 18 1 0.117400**

**"O4*" "OS" 0 1 131072 19 8 -0.354800**

**"C1*" "CT" 0 1 131072 20 6 0.039400**

**"H10" "H2" 0 1 131072 21 1 0.200700**

**"N9" "N*" 0 1 131072 22 7 -0.025100**

**"C8" "CK" 0 1 131072 23 6 0.200600**

**"H80" "H5" 0 1 131072 24 1 0.155300**

**"N7" "NB" 0 1 131072 25 7 -0.607300**

**"C5" "CB" 0 1 131072 26 6 0.051500**

**"C6" "CA" 0 1 131072 27 6 0.700900**

**"N6" "N2" 0 1 131072 28 7 -0.901900**

**"H60" "H" 0 1 131072 29 1 0.411500**

**"H61" "H" 0 1 131072 30 1 0.411500**

**"N1" "NC" 0 1 131072 31 7 -0.761500**

**"C2" "CQ" 0 1 131072 32 6 0.587500**

**"H2" "H5" 0 1 131072 33 1 0.047300**

**"N3" "NC" 0 1 131072 34 7 -0.699700**

**"C4" "CB" 0 1 131072 35 6 0.305300**

**"C3*" "CT" 0 1 131072 36 6 0.202200**

**"H30" "H1" 0 1 131072 37 1 0.061500**

**"O3*" "OH" 0 1 131072 38 8 -0.654100**

**"H3'" "HO" 0 1 131072 39 1 0.437600**

**"C2*" "CT" 0 1 131072 40 6 0.067000**

**"H20" "H1" 0 1 131072 41 1 0.097200**

**"O2*" "OH" 0 1 131072 42 8 -0.613900**

**"H2'" "HO" 0 1 131072 43 1 0.418600**

**!entry.ATP.unit.atomspertinfo table str pname str ptype int ptypex int pelmnt dbl pchg**

**"O1G" "O3" 0 -1 0.0**

**"PG" "P" 0 -1 0.0**

**"O2G" "O3" 0 -1 0.0**

**"O3G" "O3" 0 -1 0.0**

**"O3B" "OS" 0 -1 0.0**

**"PB" "P" 0 -1 0.0**

**"O1B" "O2" 0 -1 0.0**

**"O2B" "O2" 0 -1 0.0**

**"O3A" "OS" 0 -1 0.0**

**"PA" "P" 0 -1 0.0**

**"O1A" "O2" 0 -1 0.0**

**"O2A" "O2" 0 -1 0.0**

**"O5*" "OS" 0 -1 0.0**

**"C5*" "CT" 0 -1 0.0**

**"H50" "H1" 0 -1 0.0**

**"H51" "H1" 0 -1 0.0**

**"C4*" "CT" 0 -1 0.0**

**"H40" "H1" 0 -1 0.0**

**"O4*" "OS" 0 -1 0.0**

**"C1*" "CT" 0 -1 0.0**

**"H10" "H2" 0 -1 0.0**

**"N9" "N*" 0 -1 0.0**

**"C8" "CK" 0 -1 0.0**

**"H80" "H5" 0 -1 0.0**

**"N7" "NB" 0 -1 0.0**

**"C5" "CB" 0 -1 0.0**

**"C6" "CA" 0 -1 0.0**

**"N6" "N2" 0 -1 0.0**

**"H60" "H" 0 -1 0.0**

**"H61" "H" 0 -1 0.0**

**"N1" "NC" 0 -1 0.0**

**"C2" "CQ" 0 -1 0.0**

**"H2" "H5" 0 -1 0.0**

**"N3" "NC" 0 -1 0.0**

**"C4" "CB" 0 -1 0.0**

**"C3*" "CT" 0 -1 0.0**

**"H30" "H1" 0 -1 0.0**

**"O3*" "OH" 0 -1 0.0**

**"H3'" "HO" 0 -1 0.0**

**"C2*" "CT" 0 -1 0.0**

**"H20" "H1" 0 -1 0.0**

**"O2*" "OH" 0 -1 0.0**

**"H2'" "HO" 0 -1 0.0**

**!entry.ATP.unit.boundbox array dbl**

**-1.000000**

**0.0**

**0.0**

**0.0**

**0.0**

**!entry.ATP.unit.childsequence single int**

**2**

**!entry.ATP.unit.connect array int**

**0**

**40**

**!entry.ATP.unit.connectivity table int atom1x int atom2x int flags**

**42 43 1**

**40 41 1**

**40 42 1**

**38 39 1**

**36 37 1**

**36 38 1**

**36 40 1**

**34 35 1**

**32 33 1**

**32 34 1**

**31 32 1**

**28 29 1**

**28 30 1**

**27 28 1**

**27 31 1**

**26 27 1**

**26 35 1**

**25 26 1**

**23 24 1**

**23 25 1**

**22 23 1**

**22 35 1**

**20 21 1**

**20 22 1**

**20 40 1**

**19 20 1**

**17 18 1**

**17 19 1**

**17 36 1**

**14 15 1**

**14 16 1**

**14 17 1**

**13 14 1**

**10 11 1**

**10 12 1**

**10 13 1**

**9 10 1**

**6 7 1**

**6 8 1**

**6 9 1**

**5 6 1**

**2 3 1**

**2 4 1**

**2 5 1**

**1 2 1**

**!entry.ATP.unit.hierarchy table str abovetype int abovex str belowtype int belowx**

**"U" 0 "R" 1**

**"R" 1 "A" 43**

**"R" 1 "A" 42**

**"R" 1 "A" 41**

**"R" 1 "A" 40**

**"R" 1 "A" 39**

**"R" 1 "A" 38**

**"R" 1 "A" 37**

**"R" 1 "A" 36**

**"R" 1 "A" 35**

**"R" 1 "A" 34**

**"R" 1 "A" 33**

**"R" 1 "A" 32**

**"R" 1 "A" 31**

**"R" 1 "A" 30**

**"R" 1 "A" 29**

**"R" 1 "A" 28**

**"R" 1 "A" 27**

**"R" 1 "A" 26**

**"R" 1 "A" 25**

**"R" 1 "A" 24**

**"R" 1 "A" 23**

**"R" 1 "A" 22**

**"R" 1 "A" 21**

**"R" 1 "A" 20**

**"R" 1 "A" 19**

**"R" 1 "A" 18**

**"R" 1 "A" 17**

**"R" 1 "A" 16**

**"R" 1 "A" 15**

**"R" 1 "A" 14**

**"R" 1 "A" 13**

**"R" 1 "A" 12**

**"R" 1 "A" 11**

**"R" 1 "A" 10**

**"R" 1 "A" 9**

**"R" 1 "A" 8**

**"R" 1 "A" 7**

**"R" 1 "A" 6**

**"R" 1 "A" 5**

**"R" 1 "A" 4**

**"R" 1 "A" 3**

**"R" 1 "A" 2**

**"R" 1 "A" 1**

**!entry.ATP.unit.name single str**

**""**

**!entry.ATP.unit.positions table dbl x dbl y dbl z**

**83.116000 56.328000 34.799000**

**81.958000 55.946000 33.887000**

**80.622000 56.378000 34.355000**

**82.157000 56.276000 32.431000**

**81.934000 54.263000 33.995000**

**83.225000 53.200000 34.301000**

**84.025000 53.698000 35.503000**

**82.699000 51.803000 34.270000**

**84.137000 53.544000 32.913000**

**85.705000 53.961000 32.616000**

**86.143000 53.493000 31.293000**

**86.034000 55.373000 33.046000**

**86.636000 53.247000 33.851000**

**86.496000 52.322000 35.039000**

**85.528930 51.891361 35.010597**

**86.605520 52.886496 35.928286**

**87.551000 51.243000 34.972000**

**87.923832 50.920290 35.909196**

**86.964000 50.170000 34.204000**

**87.844000 49.721000 33.181000**

**87.464490 48.778726 33.480286**

**87.324000 49.788000 31.770000**

**86.307000 50.549000 31.243000**

**85.543065 51.057097 31.807066**

**86.318000 50.628000 29.929000**

**87.429000 49.837000 29.535000**

**88.021000 49.507000 28.272000**

**87.572000 49.954000 27.115000**

**88.056852 49.728976 26.269847**

**86.746767 50.517888 27.083047**

**89.091000 48.710000 28.264000**

**89.557000 48.268000 29.407000**

**90.420504 47.625841 29.343145**

**89.133000 48.487000 30.627000**

**88.035000 49.305000 30.643000**

**88.755000 51.789000 34.175000**

**88.438524 52.593635 33.563538**

**89.873000 52.207000 35.029000**

**89.628110 52.096712 35.957953**

**89.076000 50.719000 33.180000**

**89.045260 50.885193 32.134574**

**90.328000 50.093000 33.538000**

**90.804203 50.658820 34.161031**

**!entry.ATP.unit.residueconnect table int c1x int c2x int c3x int c4x int c5x int c6x**

**1 40 0 0 0 0**

**!entry.ATP.unit.residues table str name int seq int childseq int startatomx str restype int imagingx**

**"ATP" 1 44 1 "?" 0**

**!entry.ATP.unit.residuesPdbSequenceNumber array int**

**1**

**!entry.ATP.unit.solventcap array dbl**

**-1.000000**

**0.0**

**0.0**

**0.0**

**0.0**

**!entry.ATP.unit.velocities table dbl x dbl y dbl z**

**0.0 0.0 0.0**

**0.0 0.0 0.0**

**0.0 0.0 0.0**

**0.0 0.0 0.0**

**0.0 0.0 0.0**

**0.0 0.0 0.0**

**0.0 0.0 0.0**

**0.0 0.0 0.0**

**0.0 0.0 0.0**

**0.0 0.0 0.0**

**0.0 0.0 0.0**

**0.0 0.0 0.0**

**0.0 0.0 0.0**

**0.0 0.0 0.0**

**0.0 0.0 0.0**

**0.0 0.0 0.0**

**0.0 0.0 0.0**

**0.0 0.0 0.0**

**0.0 0.0 0.0**

**0.0 0.0 0.0**

**0.0 0.0 0.0**

**0.0 0.0 0.0**

**0.0 0.0 0.0**

**0.0 0.0 0.0**

**0.0 0.0 0.0**

**0.0 0.0 0.0**

**0.0 0.0 0.0**

**0.0 0.0 0.0**

**0.0 0.0 0.0**

**0.0 0.0 0.0**

**0.0 0.0 0.0**

**0.0 0.0 0.0**

**0.0 0.0 0.0**

**0.0 0.0 0.0**

**0.0 0.0 0.0**

**0.0 0.0 0.0**

**0.0 0.0 0.0**

**0.0 0.0 0.0**

**0.0 0.0 0.0**

**0.0 0.0 0.0**

**0.0 0.0 0.0**

**0.0 0.0 0.0**

**0.0 0.0 0.0**
